# Elevated target expression by dual PD-L1 and 4-1BB engagement is associated with^ 89^Zr-PD-L1x4-1BB bispecific Mabcalin tumor uptake

**DOI:** 10.7150/thno.123930

**Published:** 2026-04-22

**Authors:** Claudia A.J. van Winkel, Xiaoyu Fan, Danique Giesen, Glenn Gauderat, Steven de Jong, Thomas Jaquin, Wim Timens, Agathe Lepissier, Marleen Richter, Stefan Grüner, Nicole Andersen, Laurent Laboureur, Lucia Pattarini, Helene Lelièvre, Elisabeth G.E. de Vries, Aizea Morales-Kastresana, Marjolijn N. Lub-de Hooge

**Affiliations:** 1 Department of Medical Oncology, University Medical Center Groningen, University of Groningen; Hanzeplein 1, 9713 GZ, Groningen, the Netherlands.; 2 Department of Nuclear Medicine and Molecular Imaging, University Medical Center Groningen, University of Groningen; Hanzeplein 1, 9713 GZ, Groningen, the Netherlands.; 3 R&D Institute Servier Paris Saclay; 20 Route 128, 91190, Gif-sur-Yvette, France.; 4 Pieris Pharmaceuticals; Zeppelinstraße 3, 85399, Hallbergmoos, Germany.; 5 Department of Pathology and Medical Biology, University Medical Center Groningen, University of Groningen; Hanzeplein 1, 9713 GZ, Groningen, the Netherlands.; 6 Department of Clinical Pharmacy and Pharmacology, University Medical Center Groningen, University of Groningen; Hanzeplein 1, 9713 GZ, Groningen, the Netherlands.

**Keywords:** cancer immunotherapy, bispecific antibodies, biodistribution, PET imaging, pharmacokinetics

## Abstract

The antibody-anticalin fusion protein (Mabcalin) targeting programmed cell death-ligand 1 (PD-L1) and T-cell costimulatory immunoreceptor CD137 (4-1BB) is designed to enhance T-cell reactivity while preventing T-cell inhibition by PD-L1/programmed cell death protein 1 (PD-1) checkpoint blockade. Using positron emission tomography (PET) imaging and *ex vivo* analysis, we investigated the factors influencing biodistribution, tumor uptake, and the influence of dose and target presence.

**Methods** Murine or human PD-L1-reactive and human 4-1BB reactive Mabcalins (mPD-L1xh4-1BB and hPD-L1xh4-1BB) were generated, radiolabeled with ^89^Zr, and administered to human 4-1BB knock-in (h4-1BB KI) or wild-type (WT) mice bearing PD-L1-positive mouse MC38 tumors. Mice underwent serial PET imaging on days 1, 2, and 4 or on days 2, 4, and 7 post intravenous injection, followed by *ex vivo* biodistribution. The intratumoral distribution of 2 protein doses of ^89^Zr-radiolabeled mPD-L1xh4-1BB and hPD-L1xh4-1BB was examined using autoradiography on tumor tissue sections. These tumor sections were immunohistochemically stained for PD-L1, CD3, CD8, and 4-1BB to link uptake to target expression levels.

**Results** ^89^Zr-mPD-L1xh4-1BB, able to bind mPD-L1 and h4-1BB in h4-1BB KI mice, predominantly showed specific and rapid dose-dependent lymphoid tissue uptake. The tumor uptake of 200 µg ^89^Zr-mPD-L1xh4-1BB in h4-1BB KI mice was also specific and increased over time. Tumor uptake in this group, where both targets PD-L1 and 4-1BB could be bound, was > 4-fold higher than in the groups that could bind only PD-L1 or 4-1BB. Dual PD-L1 and 4-1BB Mabcalin engagement at a therapeutic dose also resulted in elevated tumor protein expression levels for PD-L1, CD3, and CD8, which were lower when only PD-L1 or 4-1BB was engaged. The lowest expression was observed with the Mabcalin binding non-specifically (*P*_trend_≤0.01 for PD-L1 and CD3, *P*_trend_≤0.05 for CD8).

**Conclusion** The biodistribution of mPD-L1xh4-1BB is specific, dose-dependent, and associated with the elevated target expression resulting from dual PD-L1 and 4-1BB engagement.

## Introduction

The impact of immune checkpoint inhibitors disrupting the programmed cell death protein 1 (PD-1) and programmed cell death-ligand 1 (PD-L1) interaction has fundamentally improved treatment options for patients with a wide range of solid tumor types 1. However, many patients either fail to respond to this treatment or respond only transiently 2. Therefore, novel drug constructs and targets are continuously researched to improve response rates and durability of response. Among these, agonistic antibodies targeting the T-cell costimulatory immunoreceptor CD137 (4-1BB), such as urelumab, have shown anticancer efficacy in mouse models. However, their clinical development is hindered by liver toxicity caused by the activation of 4-1BB-positive liver-infiltrating T-cells 3. Bispecific antibodies targeting PD-L1 and 4-1BB aim to focus 4-1BB agonism mainly to PD-L1-positive tissues to mitigate 4-1BB-related liver toxicity while enhancing therapeutic efficacy compared to PD-L1 checkpoint inhibition alone 4. Several of these bispecific antibodies with different formats are in clinical development, with one already demonstrating antitumoral activity in a refractory setting 5.

**Figure 1:**
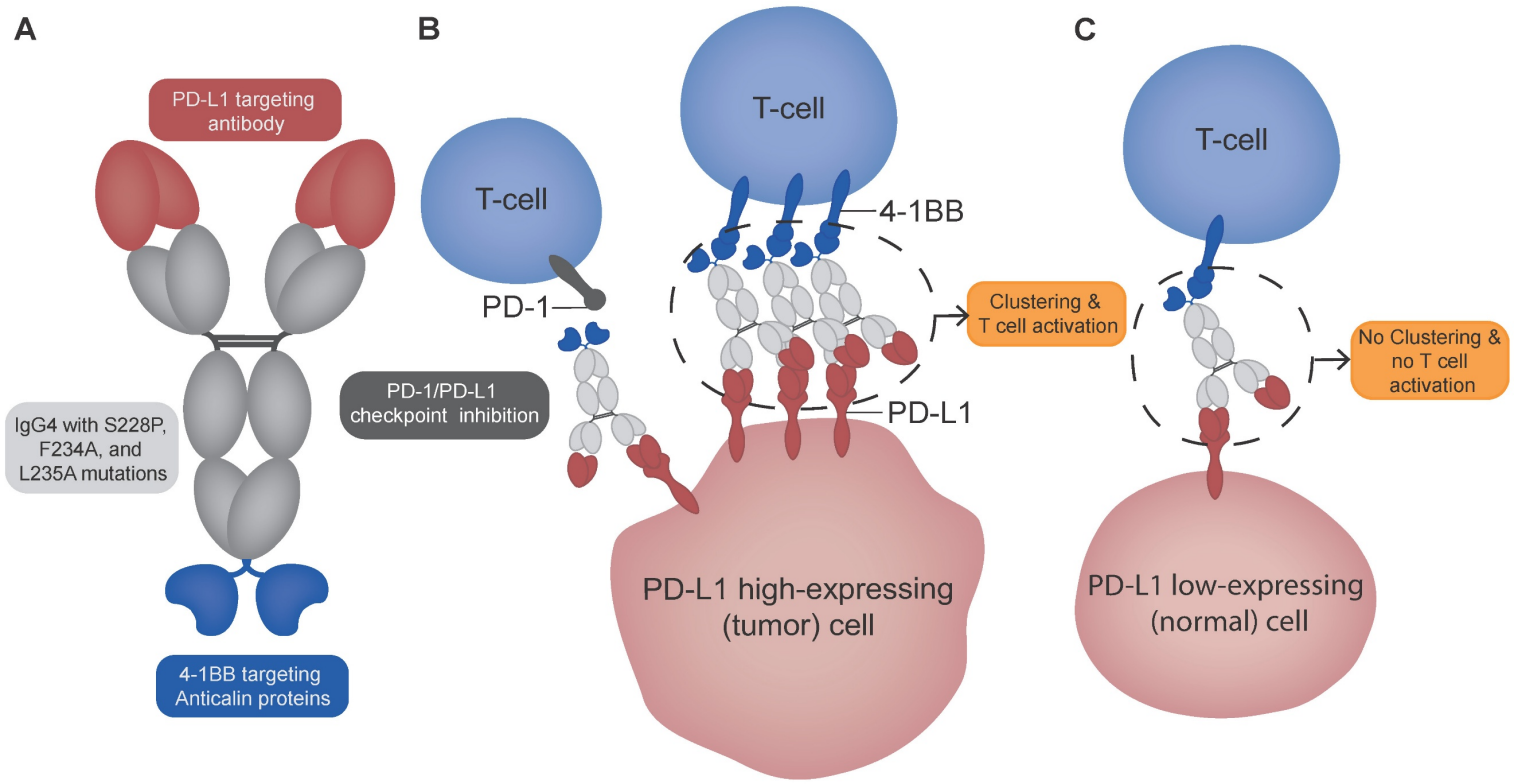
** Schematic overview of PD-L1x4-1BB Mabcalin structure and suggested mechanism of action. (A)** The PD-L1x4-1BB Mabcalin consists of a PD-L1-targeting IgG4 antibody fused with two 4-1BB-targeting anticalin proteins. The IgG4 backbone contains hinge stabilization mutation S228P and F234A and L235A mutations to reduce Fc gamma receptor binding while maintaining neonatal Fc receptor binding. **(B)** The dual binding design allows 4-1BB clustering and subsequent T-cell costimulation in the presence of PD-L1 high-expressing (tumor) cells. In addition, the PD-L1x4-1BB Mabcalin acts by blocking the PD-1/PD-L1 checkpoint. **(C)** In contrast, in PD-L1 low-expressing (normal) cells, the 4-1BB clustering is prevented, therefore not resulting in T-cell costimulation. The Figure was designed using Adobe Illustrator version 28.5.

The “2+2” tetravalent anti-human PD-L1 and 4-1BB targeting antibody-anticalin fusion protein (Mabcalin) was designed with a seven-fold higher relative affinity for PD-L1 compared to 4-1BB with a dissociation constant of 0.68 ± 0.14 nM for PD-L1 and 4.84 ± 0.24 nM for 4-1BB (Figure 1) 6, 7. In vitro studies showed stimulation of T-cells through PD-L1/PD-1 checkpoint inhibition and potent 4-1BB costimulation in a PD-L1-dependent manner 6. In human 4-1BB knock-in C57BL/6 (h4-1BB KI) mice bearing human PD-L1 positive tumors, fully human reactive PD-L1x4-1BB Mabcalin induced a stronger tumor response than the parental bivalent PD-L1 antibody 6. A clinical trial with this Mabcalin was initiated to test its safety and antitumor activity (NCT05159388).

As for all multispecific antibodies, the bispecific nature of this PD-L1x4-1BB Mabcalin makes its biodistribution hard to predict 8. It will most likely differ from the biodistribution mediated solely by 4-1BB or PD-L1 target engagement, also because PD-L1 is dynamically expressed on tumor cells, tumor-associated macrophages, and other immune cells in and outside the tumor 9.

The biodistribution of antibodies can be studied *in vivo* with molecular imaging by radiolabeling the antibody with a radionuclide and using single photon emission computed tomography (SPECT) or positron emission tomography (PET). These imaging techniques can provide quantitative spatial and temporal information on the antibody's biodistribution, including tumor tissue and target expression 10. Such biodistribution data of multispecific antibodies can provide valuable insight for making early go/no-go decisions during drug development 8, 11. For example, a SPECT study with ^111^In-labeled anti-human epidermal growth factor receptor 2 (HER2) and cluster differentiation 3 (CD3) bispecific antibody showed CD3-dependent tumor uptake in human CD3 transgenic mice bearing human HER2-transfected murine tumors. Higher CD3 affinity resulted in lower HER2-dependent tumor uptake and higher uptake in T-cell-containing lymphoid tissues 12.

PET radionuclides are often used for preclinical and clinical studies, given the high resolution of PET cameras. The PET radionuclide zirconium-89 (^89^Zr; T_½_ = 78.4 h) is especially suited for radiolabeled antibodies, as its physical half-life matches the time antibodies require for tissue distribution 13.

This study aimed to investigate the dose and target-mediated biodistribution and tumor uptake of the bispecific PD-L1x4-1BB Mabcalin using preclinical PET imaging and *ex vivo* analysis. For this purpose, we used two different ^89^Zr-labeled Mabcalins: one targeting murine PD-L1 and human 4-1BB 1BB (mPD-L1xh4-1BB), and the other targeting human PD-L1 and human 4-1BB (hPD-L1xh4-1BB). The two Mabcalins were studied at both low and therapeutic doses in two mouse models, namely human 4-1BB knock-in or wild-type (WT) mice bearing PD-L1-positive murine colon adenocarcinoma (MC38) tumors. The experiment design, with two molecules administered at a low and therapeutic dose in two mouse models with and without human 4-1BB presence, allowed us to unravel the dose- and target-mediated distribution.

## Methods

### Characterization, conjugation, and ^89^Zr-radiolabeling of PD-L1x4-1BB Mabcalins

To study the dual binding to PD-L1 and 4-1BB, and single binding to each target separately, the biodistribution was assessed with two ^89^Zr-labeled PD-L1x4-1BB Mabcalins: mPD-L1xh4-1BB targeting murine PD-L1 and human 4-1BB, and hPD-L1xh4-1BB targeting human PD-L1 and human 4-1BB (hPD-L1xh4-1BB is also known as S095012 and PRS-344). The Mabcalins were codeveloped by Servier and Pieris Pharmaceuticals. The binding affinities of the Mabcalins for PD-L1 and 4-1BB were evaluated using a dual-binding enzyme-linked immunosorbent assay (ELISA), as previously described 7. Briefly, recombinant human 4-1BB protein was coated onto the ELISA plates. The bispecific Mabcalins were then added, allowing the human 4-1BB-binding arm to engage with the coated protein. Detection was performed using biotinylated human or murine PD-L1-Fc and extravidin-horseradish peroxidase (HRP). This setup ensures that a signal is only generated when Mabcalin binds both targets, 4-1BB on the plate and PD-L1 in solution, thus confirming dual specificity.

Both antibodies, which target human PD-L1 with comparable affinities (Figure S1), were fused to anti-human 4-1BB anticalin proteins, and contained a human IgG4 backbone with an S228P hinge-stabilizing mutation, as well as F234A and L235A mutations (EU-numbering) to reduce interaction with Fc-gamma receptors. The conjugation to the bifunctional chelator DFO (Macrocyclics, B-705), ^89^Zr-radiolabeling, characterization, and quality control of both PD-L1x4-1BB Mabcalins were performed as described earlier 14, and are detailed in the supplemental materials.

### Internalization of hPD-L1xh4-1BB in human PD-L1 expressing tumor cell lines and stimulated human T-cells

A flow cytometry-based method was developed to quantitatively measure the internalization of hPD-L1xh4-1BB in tumor and immune cells. This method was developed because conventional antibody stripping techniques were unsuitable for immune cells, and microscopy does not provide quantitative data. hPD-L1xh4-1BB was biotinylated and subsequently fluorescently labeled with Alexa Fluor 647^TM^ (AF647) streptavidin (Invitrogen, #S32357). To mimic the internalization properties of ^89^Zr-DFO-hPD-L1xh4-1BB, the hPD-L1xh4-1BB bispecific was biotinylated with 5 times molar excess of NHS-biotin (EZ-Link^TM^; Thermo Scientific, #21425), as it reacts similarly to DFO with primary amines of lysine residues in the Mabcalin. Quality control of biotinylated hPD-L1xh4-1BB was similarly performed as for the DFO-conjugated Mabcalin (Figure S2A-D). IgG4 (Sigma-Aldrich, #I4640) was also biotinylated and served as a control to assess non-specific internalization. Three tumor cell lines were used, namely PD-L1 knock-out human ovarian clear cell carcinoma ES2, wild-type ES2, and triple-negative breast cancer MDA-MB-231 cell lines, representing no, high, and moderate PD-L1 expression, respectively (Figure S4A) 15. PBMCs were isolated from three healthy donors' blood buffer coats (Sanquin, the Netherlands) with Ficoll-Paque^TM^ plus (Cytiva, #17144002). To induce PD-L1 and 4-1BB expression on PBMCs, a cell stimulation cocktail containing ionomycin and phorbol 12-myristate 13-acetate (PMA; eBioscience, #00-4970-93) for 16 h, or recombinant interleukin-2 (IL-2, Novartis) and lectin from Phaseolus vulgaris (PHA; Sigma-Aldrich, #L8754) for 3 days, was added as described earlier 16. 4-1BB and PD-L1 expressions on stimulated T-cells derived from PBMCs were determined with the antibodies APC-4-1BB (clone 4B4-1, BD Biosciences, #550890) and APC-PD-L1 (clone 29E.2A3, BD Biosciences, #568315). Ionomycin and PMA stimulation induced PD-L1 and 4-1BB, whereas IL-2 and PHA stimulation induced only PD-L1, and to a lower level than induced by ionomycin and PMA (Figure S4B-C).

**Figure 2:**
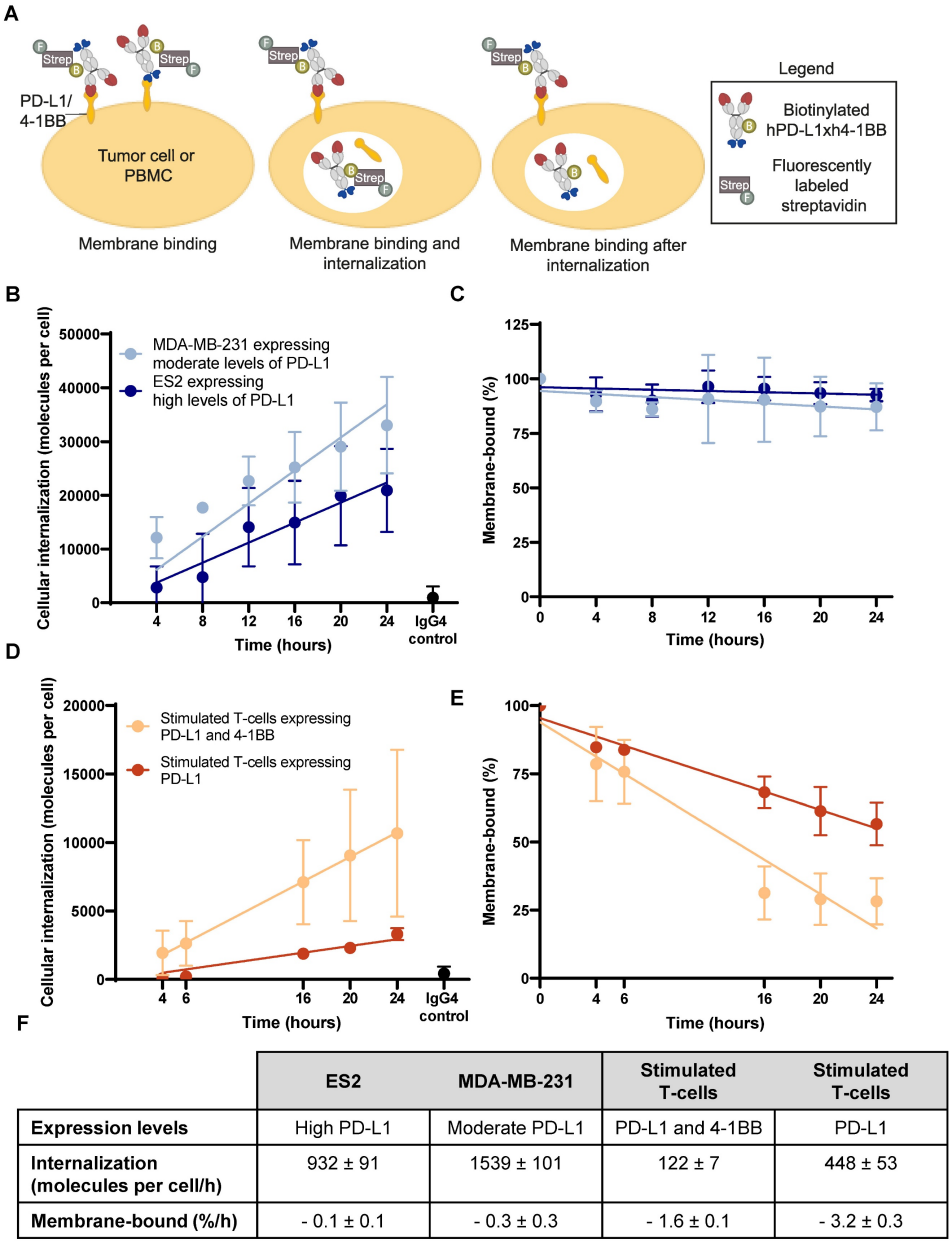
** Internalization of biotinylated hPD-L1xh4-1BB in PD-L1 expressing human tumor cells and stimulated T-cells. (A)** Schematic representation of the experimental set-up to quantify biotinylated hPD-L1xh4-1BB internalization with Alexa Fluor 647^TM^ labeled streptavidin. The hPD-L1xh4-1BB internalization degree was assessed by subtracting the membrane binding together with the internalized fraction (middle image) from the membrane binding after internalization (right image). The baseline membrane binding (left image) was used as a reference for expressing membrane binding as a percentage. **(B)** Internalization of biotinylated hPD-L1xh4-1BB in PD-L1 expressing human tumor cell lines ES2 (n=4), MDA-MB-231 (n=3), and PD-L1 knock-out ES2 (n=3) determined with flow cytometry and quantified in molecules per cell with quantification beads. The PD-L1 knock-out ES2 and IgG4 control reflects antibody internalization after an 24-hour incubation. **(C)** Percentage membrane-bound biotinylated hPD-L1xh4-1BB on tumor cells after incubation. **(D)** Internalization of biotinylated hPD-L1xh4-1BB in stimulated human T-cells expressing PD-L1 and/or 4-1BB (n=3) expressed as molecules per cell. IgG4 control reflects antibody internalization after an 24-hour incubation. **(E)** Percentage membrane-bound biotinylated hPD-L1xh4-1BB on stimulated human T-cells. **(F)** Table summarizing the 4-1BB and PD-L1 expression levels, internalization per h (molecules per cell/h), and membrane binding after internalization (%/h) of biotinylated hPD-L1xh4-1BB in ES2, MDA-MB-231, and stimulated human T-cells expressing PD-L1 and/or 4-1BB. Data are presented as mean ± standard deviation. %: percentage; ; ES2 PD-L1 KO: PD-L1 knock-out ES2 cells; Strep: streptavidin.

To determine internalized hPD-L1xh4-1BB, AF647-streptavidin was added to the cells at different time points directly with the biotinylated hPD-L1xh4-1BB for 37°C incubation, or when the cells were cooled on ice to stain membrane-bound biotinylated hPD-L1xh4-1BB (Figure 2A). PBMCs, stimulated PBMCs (n=3), MDA-MB-231 (n=3), ES2 (n=4), PD-L1 knock-out ES2 (n=3) cells were seeded in triplicate in a 48-well plate with 1 x 10^5^ cells per well, followed by a human Fc block (BD Biosciences, #564219) for PBMCs. Biotinylated hPD-L1xh4-1BB at 10 µg/mL was added and incubated for 4, 6, 8, 12, 16, 20, or 24 h at 37°C and 5% CO_2_. The biotinylated IgG4 control at 10 µg/mL and biotinylated hPD-L1xh4-1BB were incubated for 24 h. In the cells where internalization and membrane-binding were measured, 10 µg/mL AF647-streptavidin was added simultaneously with biotinylated hPD-L1xh4-1BB and incubated for the indicated time at 37 °C (Figure 2A). After incubation, cells were washed three times with 5% fetal calf serum in phosphate-buffered saline (PBS; 150 mM NaCl, 10 mM Na_2_HPO_4_, 1.6 mM KH_2_PO_4_, pH 7.3) and cooled on ice. In the cells where only membrane-bound biotinylated hPD-L1xh4-1BB was measured, 10 µg/mL AF647-streptavidin was added and stained for 30 min on ice (Figure 2A). PBMCs and stimulated PBMCs were simultaneously stained with BV421-CD3 antibody (clone SK7, BD Biosciences, #563798). After three washing cycles, cell dissociation buffer (Gibco, #13151014) was added for 10 min at 37°C to adherent tumor cells. Cell viability was determined with propidium iodide (Invitrogen, #P1304MP) and measured with NovoCyte Quanteon 4025 (Agilent). Data analysis was performed with NovoExpress flow cytometry software version 1.6.1. The AF647 mean fluorescence intensity (MFI) within live single tumor cells and T-cells served to detect membrane-bound and internalized biotinylated hPD-L1xh4-1BB (Figure S5). The MFI was quantified in molecules per cell with Quantum Simply Cellular anti-human IgG beads (Bangs Laboratories, #816). Internalization per h (molecules per cell/h) and membrane binding after internalization (%/h) were calculated with simple linear regression in GraphPad Prism version 9.1.0.

### PET imaging and *ex vivo* tissue uptake of ^89^Zr-labeled PD-L1x4-1BB Mabcalins in murine tumor-bearing mice

The Institutional Animal Care and Use Committee of the University of Groningen, the Netherlands, approved the animal PET experiments (#2010686-01-002). Studies were performed in two mouse strains, namely PD-L1-positive murine tumor-bearing h4-1BB KI and WT C57BL/6 mice.

h4-1BB KI mice (developed by and purchased from Biocytogen, #110004) are genetically engineered mice in which the h4-1BB extracellular domain is knocked in to replace the murine 4-1BB extracellular domain. After an acclimatization period of two weeks, MC38 cells (1.5 x 10^6^) that endogenously express PD-L1 were implanted subcutaneously in the right flank of 8 to 12-week-old female h4-1BB KI and WT mice 17. Mice (sample size 4-7 per group, based on a power analysis; 37 in total) were assigned to groups based on their tumor burden to ensure similarity in tumor sizes before tracer injection. The primary investigator was aware of the group allocation, and blinding was not necessary due to the measurable nature of the data. All mice that completed the predetermined experimental plan in compliance with Institutional Animal Care and Use Committee (IACUC) guidelines were included for data analysis. No mice, outliers, or other data points were excluded. Tumor size was monitored three times per week with caliper measurements, and tumors were grown to a minimum of ≥ 50 mm^3^ before tracer administration.

h4-1BB KI and WT C57BL/6 tumor-bearing mice received ^89^Zr-mPD-L1xh4-1BB or ^89^Zr-hPD-L1xh4-1BB to distinguish between binding to PD-L1 and 4-1BB, PD-L1 only, 4-1BB only, or non-specific binding (Figures 4B and 5B, Figures S6A and S7A). ^89^Zr-mPD-L1xh4-1BB, when given to h4-1BB KI mice, could bind to both mPD-L1 and h4-1BB, as these mice have both targets present. ^89^Zr-hPD-L1xh4-1BB in h4-1BB KI mice can bind to h4-1BB only, given that only human 4-1BB is present in these mice, human PD-L1 is absent, and the bispecific is not cross-reactive. ^89^Zr-mPD-L1xh4-1BB in WT mice can only bind to murine PD-L1 due to the absence of human 4-1BB in these mice (Figure 5B). In WT mice, ^89^Zr-hPD-L1xh4-1BB can only bind non-specifically as none of its targets are present (Figures S6A and S7A).

Two protein doses of 30 µg and 200 µg were chosen based on the pharmacological effects of mPD-L1xh4-1BB in h4-1BB KI mice 7. The low dose of 30 µg is subtherapeutic and suppresses tumor growth, the higher 200 µg dose is considered therapeutic and reduces tumor size (Figure S8) 7. For the 30 µg dose groups, WT or h4-1BB KI mice received retro-orbitally intravenously 30 µg ^89^Zr-DFO-mPD-L1xh4-1BB or ^89^Zr-DFO-hPD-L1xh4-1BB labeled with 2.5 (83 MBq/mg) or 5 MBq (167 MBq/mg) ^89^Zr for respectively imaging up to 4 or 7 days post-injection. For the 200 µg dose groups, the 30 µg protein dose was supplemented up to 200 µg with mPD-L1xh4-1BB or hPD-L1xh4-1BB. Serial PET imaging was performed on days 1, 2, and 4, or on days 2, 4, and 7 post-tracer injection with a Focus 220 rodent scanner (CTI Siemens; Figures 4A and 5A). The *in vivo* and *ex vivo* biodistribution were normalized to the injected activity in standard uptake value (SUV) and percentage injected dose per gram (%ID/g) to normalize for the two different specific activities used. Total radioactivity in the mice was measured with a dose calibrator directly after injection and on the days of PET imaging.

**Figure 3:**
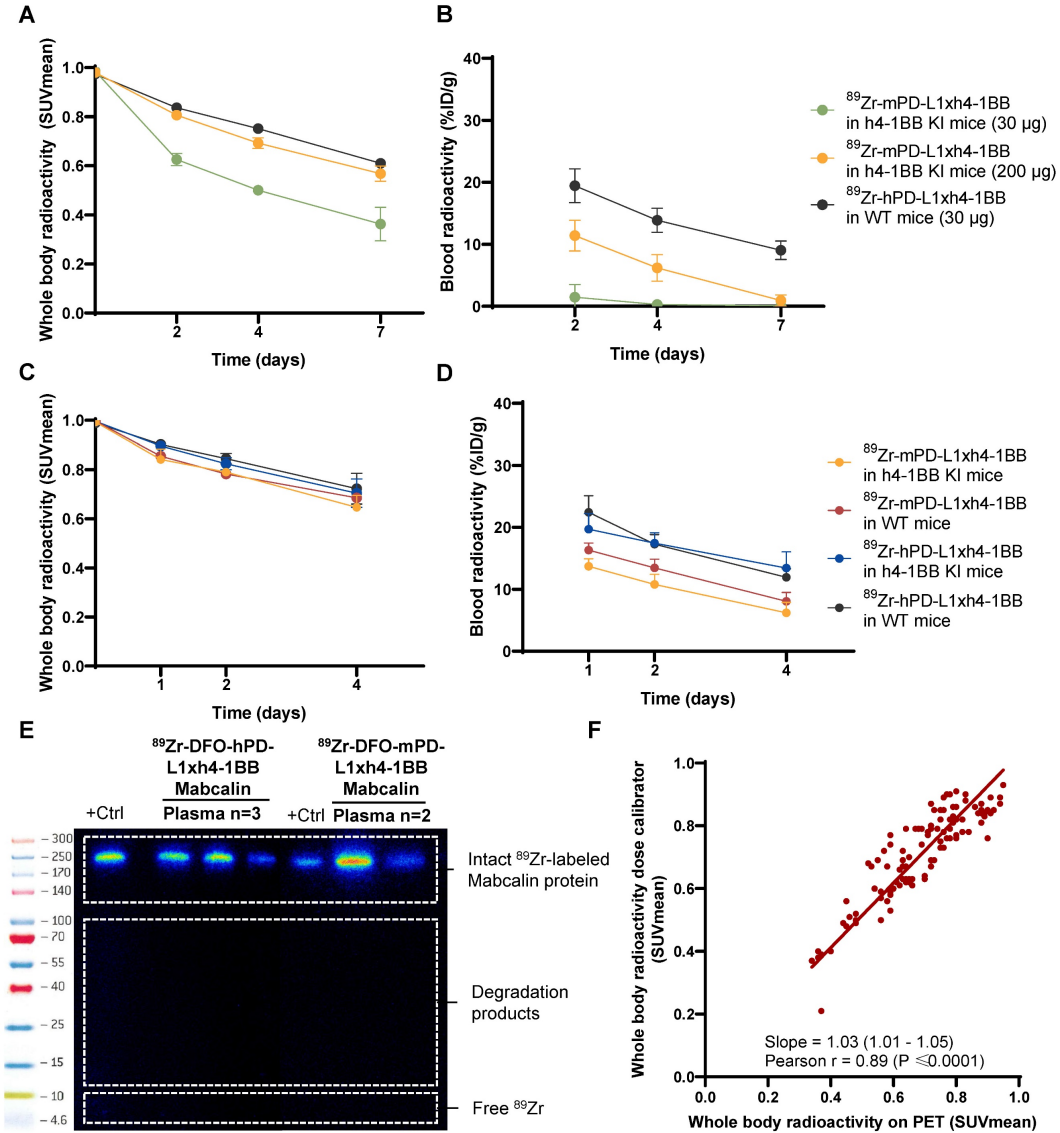
** Radioactivity in whole-body and whole blood and plasma tracer integrity of ^89^Zr-mPD-L1xh4-1BB and ^89^Zr-hPD-L1xh4-1BB. (A)** ^89^Zr-radioactivity in whole-body of mPD-L1xh4-1BB and hPD-L1xh4-1BB in h4-1BB KI or WT mice dosed at 30 or 200 µg at days 2, 4, and 7 post-injection. **(B)**^ 89^Zr-radioactivity in whole blood of mPD-L1xh4-1BB and hPD-L1xh4-1BB in h4-1BB KI or WT mice dosed at 30 or 200 µg at days 2, 4, and 7 post-injection. **(C)** ^89^Zr-radioactivity in whole-body of ^89^Zr-mPD-L1xh4-1BB and ^89^Zr-hPD-L1xh4-1BB in h4-1BB KI or WT mice dosed at 200 µg at days 1, 2, and 4 post-injection. **(D)** ^89^Zr-radioactivity in whole blood of mPD-L1xh4-1BB and hPD-L1xh4-1BB in h4-1BB KI or WT mice dosed at 200 µg at days 1, 2, and 4 post-injection. **(E)** *Ex vivo* tracer integrity of ^89^Zr-mPD-L1xh4-1BB or ^89^Zr-hPD-L1xh4-1BB in plasma samples 4 days post-injection, as determined by SDS-PAGE. Detection of the signal was performed with autoradiography. Tracer integrity was also determined 7 days post-injection and the same results were obtained. **(F)** Correlation between whole-body SUVmean values measured by PET and *ex vivo* in a dose calibrator. Data are presented as mean ± standard deviation. %ID/g: percentage injected dose per g; Ctrl: control; h: human; h4-1BB KI mice: human 4-1BB knock-in mice; m: murine; SUV: standard uptake value; WT mice: wild-type mice.

PET data were reconstructed and analyzed using AMIDE medical image data examiner software version 1.0.4. The accuracy of the PET data was validated based on the correlation between the whole body mean standard uptake value (SUVmean) measured by PET and in the dose calibrator (Figure 3F). Tracer presence in the blood pool (i.e., in the heart) was quantified in a size-fixed sphere drawn in the heart and expressed as SUVmean, with a detection limit set at twice the whole body SUVmean. The tumor maximum standard uptake value (SUVmax) was measured in a three-dimensional region around the tumor. Tracer tumor uptake was expressed as a tumor-to-blood ratio (SUVmax/SUVmean) to correct for exposure differences in blood between experimental mouse groups (Figure 3A-D). If the heart SUVmean was below the detection limit, radioactivity in *ex vivo* blood samples obtained as described below was used and expressed as SUVmean.

After each PET scan, blood samples were collected in lithium-heparin tubes (MiniCollect Tube 0.8mL separator, Greiner) for tracer pharmacokinetics (PK) via retro-orbital blood collection. Tracer integrity was tested in the plasma samples collected after the last PET scan (supplemental methods). Radioactivity in the whole blood and plasma samples was measured in a calibrated Wizard γ-counter (Wizard2 2480; PerkinElmer). The counts were converted into a percentage injected dose (%ID) using a standard curve of injected tracer and expressed per weight (g). The whole blood exposure (%ID/g*h), defined as the area under the time activity curve (AUC) over the time frame of PET imaging, was calculated with non-compartmental PK analysis in the ubiquity package version 2.0.0 in R version 4.3.1 and Rstudio version 2023.06.1.

After the last PET scan on either days 4 or 7, mice were sacrificed to extract relevant tissue for *ex vivo* analysis. Several lymph nodes from various anatomical locations, including cervical, axillary, and mesenteric, were collected. Tumor-draining lymph nodes were identified as those either situated on the tumor or within the same subcutaneous skin layer as the tumor. Tumors and organs of interest were collected, weighed, and measured in a calibrated Wizard γ-counter. Tissue tracer uptake was expressed as %ID/g and tissue-to-whole blood AUC (AUC_wb_) ratio for tissues expressing murine PD-L1 and/or human or murine 4-1BB, including bone marrow, spleen, tumor, mesenteric, axillary, cervical, and tumor-draining lymph nodes. The ratio was chosen because the antibody internalizes upon target binding, followed by ^89^Zr-residualization. This is influenced by the tracer exposure over the imaging time frame. Tissues were formalin-fixed and paraffin-embedded for autoradiography (supplemental methods) and histology and immunohistochemistry (IHC) analysis.

### *Ex vivo* tumor tissue analyses

To evaluate the tumor microenvironment after mPD-L1xh4-1BB or hPD-L1xh4-1BB administration, MC38 tumor sections were stained with hematoxylin and eosin (H&E) and immunohistochemically for murine PD-L1, CD3, and CD8. H&E staining was performed on tumor sections used for autoradiography, and IHC staining on consecutive 4 µm sections. Tumors of h4-1BB KI mice were also stained for human 4-1BB. The staining procedure for murine PD-L1 is performed as described previously 17. Stainings were performed with 1:100 anti-CD3 (clone CD3-12, Abcam, #ab11089), 1:1000 anti-CD8 (clone EPR21769, Abcam, #ab217344), or 1:100 anti-4-1BB (clone E6Z7F, Cell Signaling Technology, #19541) after antigen-retrieval with 10 nM Tris/EDTA buffer (pH 9.0). Secondary antibody Envision+System-HRP anti-rabbit (Dako, #K4003) was applied for CD3 and 4-1BB, and ImmPRESS polymer anti-rat IgG (Vector Laboratories, #MP-7404-50) for CD8, followed by diaminobenzidine chromogen (DAB) and hematoxylin counterstaining. Digital scans of slides were acquired by a NanoZoomer 2.0-HT multi-slide scanner (Hamamatsu). The percentage of DAB-stained area was quantified across the entire tumor section for all tumors in all experiment groups using positive pixel counting in Qupath version 0.5.0 18. Given the limited number of mice per group, expression levels of PD-L1, CD3, and CD8 on days 4 and 7 were aggregated for h4-1BB KI mice dosed with 200 µg mPD-L1xh4-1BB, in which a therapeutic effect was anticipated. Similarly, tumor expression data on days 4 and 7 from WT mice treated with hPD-L1xh4-1BB were also combined, given the absence of antitumor effects in these tumors. Data were also pooled from mPD-L1xh4-1BB-treated WT mice and hPD-L1xh4-1BB-treated h4-1BB KI mice, which each engage only PD-L1 or only 4-1BB. Finally, 4-1BB tumor expression levels were compared between h4-1BB KI mice that received 200 µg mPD-L1xh4-1BB and hPD-L1xh4-1BB, as the hPD-L1xh4-1BB in this mouse model is not expected to induce a therapeutic effect.

### Statistical analysis

Data are presented as mean ± standard deviation and median ± interquartile range as indicated in the Figure legends. Statistical analyses were performed in GraphPad Prism version 9.1.0. using the Mann-Whitney U test (two groups), a Kruskal-Wallis test followed by Dunn's multiple comparison test (> 2 groups), or a Pearson correlation test. Trends were tested with Cuzick's test in Rstudio version 2023.06.1 and R version 4.3 19. *P*-values ˂ 0.05 were considered significant.

## Results

### Internalization of hPD-L1xh4-1BB in human tumor cells and stimulated T-cells

The extent of ^89^Zr-PD-L1x4-1BB Mabcalin internalization is determined to better interpret tissue radioactivity uptake, as ^89^Zr can residualize intracellularly and accumulate in target tissues. Using flow cytometry, the internalization of biotinylated hPD-L1xh4-1BB, as surrogate for p-SCN-Bn-deferoxamine (DFO)-conjugated Mabcalin, was assessed in three settings: in two PD-L1 expressing human tumor cell lines and in human peripheral blood mononuclear cells (PBMC)-derived T-cells stimulated to express PD-L1 and/or 4-1BB (Figure 2A, Figures S1A, S2, and S4). Internalization dynamics differed between tumor cells and T-cells. Biotinylated hPD-L1xh4-1BB internalized in both tumor cell lines at similar rates (Figure 2B, F). After internalization, the membrane-bound biotinylated hPD-L1xh4-1BB fraction remained constant, as most likely internalized PD-L1 receptors could be recycled or newly expressed. The hPD-L1xh4-1BB excess in the supernatant can bind these receptors and undergo subsequent internalization (Figure 2C, F). Our results indicated that the internalization rate of PD-L1 receptors was similar to the membrane PD-L1 re-expression and/or synthesis rate. However, in stimulated human T-cells expressing PD-L1 and 4-1BB, the internalization rate was 3.7-fold higher than in T-cells expressing only PD-L1 (Figure 2D, F). The membrane-bound biotinylated hPD-L1xh4-1BB fraction on stimulated human T-cells diminished after hPD-L1xh4-1BB binding and PD-L1 and/or 4-1BB target internalization, resulting in decreased target availability on the cell membrane. The results thus suggest lower target recycling and/or *de novo* target synthesis by T-cells than by tumor cells (Figure 2E-F).

### Specific tumor and lymphoid tissue uptake of the ^89^Zr-mPD-L1xh4-1BB in MC38 tumor-bearing h4-1BB KI mice

mPD-L1xh4-1BB and hPD-L1xh4-1BB were DFO-conjugated and ^89^Zr-radiolabeled, achieving ≥ 95% radiochemical and protein purity, with ≤ 5% radioactive and protein aggregates and fragments. Their binding affinity and potency were preserved. Despite a minor shift in affinity, the 4-1BB binding domain retained its functional integrity. Therefore, any effect of DFO conjugation on 4-1BB-mediated uptake was considered minimal within the context of this study (Figures S1A-B and S3C-M). The pharmacokinetics remained unchanged in CD-1 mice (S3N, O).

Specific tumor and tissue uptake of PD-L1x4-1B Mabcalin was evaluated with ^89^Zr-mPD-L1xh4-1BB in h4-1BB KI mice, expressing endogenous murine PD-L1 and human 4-1BB. Uptake was compared to the non-specific uptake of ^89^Zr-hPD-L1xh4-1BB in WT mice, not expressing human PD-L1 and 4-1BB (Figures S6A and S7A). Both h4-1BB KI and WT mice were implanted with MC38 tumors endogenously expressing murine PD-L1 (Figure S1C).

PET imaging revealed higher tumor-to-blood ratios for ^89^Zr-mPD-L1xh4-1BB in h4-1BB KI mice than for ^89^Zr-hPD-L1xh4-1BB in WT mice bearing MC38 tumors, both at the 30 µg protein dose on days 2, 4, and 7 and at the 200 µg protein dose on days 1, 2, and 4 (Figures S6B, C and S7B, C). This indicates specific tumor uptake of ^89^Zr-mPD-L1xh4-1BB, which can bind mPD-L1 and h4-1BB in the h4-1BB KI mice, compared to non-specific uptake of ^89^Zr-hPD-L1xh4-1BB in WT mice, lacking its targets. *Ex vivo* analysis confirmed specific ^89^Zr-mPD-L1xh4-1BB tumor uptake in h4-1BB KI mice with higher tumor-to-AUC_WB_, _0-4 days/0-7 days_ ratios than for ^89^Zr-hPD-L1xh4-1BB in WT mice (Figures S6D and S7D). Also, in lymphoid tissues, including bone marrow, thymus, spleen, mesenteric, cervical, and tumor-draining lymph nodes, specific uptake is found for the 30 µg and 200 µg dose groups (Figures S6D and S6D). At a 200 µg protein dose, higher ^89^Zr-mPD-L1xh4-1BB tissue uptake was also observed in the lung, liver, and stomach of h4-1BB KI mice (Figure S7E). The 30 µg ^89^Zr-mPD-L1xh4-1BB protein dose in h4-1BB KI mice was excreted and cleared from the blood faster than the non-specific 30 µg ^89^Zr-hPD-L1xh4-1BB control in WT mice (Figure 3A-B). This rapid clearance of the low dose was likely due to target-mediated drug disposition (TMDD) in lymphoid tissues. It also resulted in low absolute ^89^Zr-mPD-L1xh4-1BB uptake in the heart, urine, bladder, kidney, stomach, colon, muscle, skin, and brain in h4-1BB KI mice (Figure S6E).

### Dose-dependent biodistribution of the ^89^Zr-mPD-L1xh4-1BB in MC38 tumor-bearing h4-1BB KI mice

**Figure 4:**
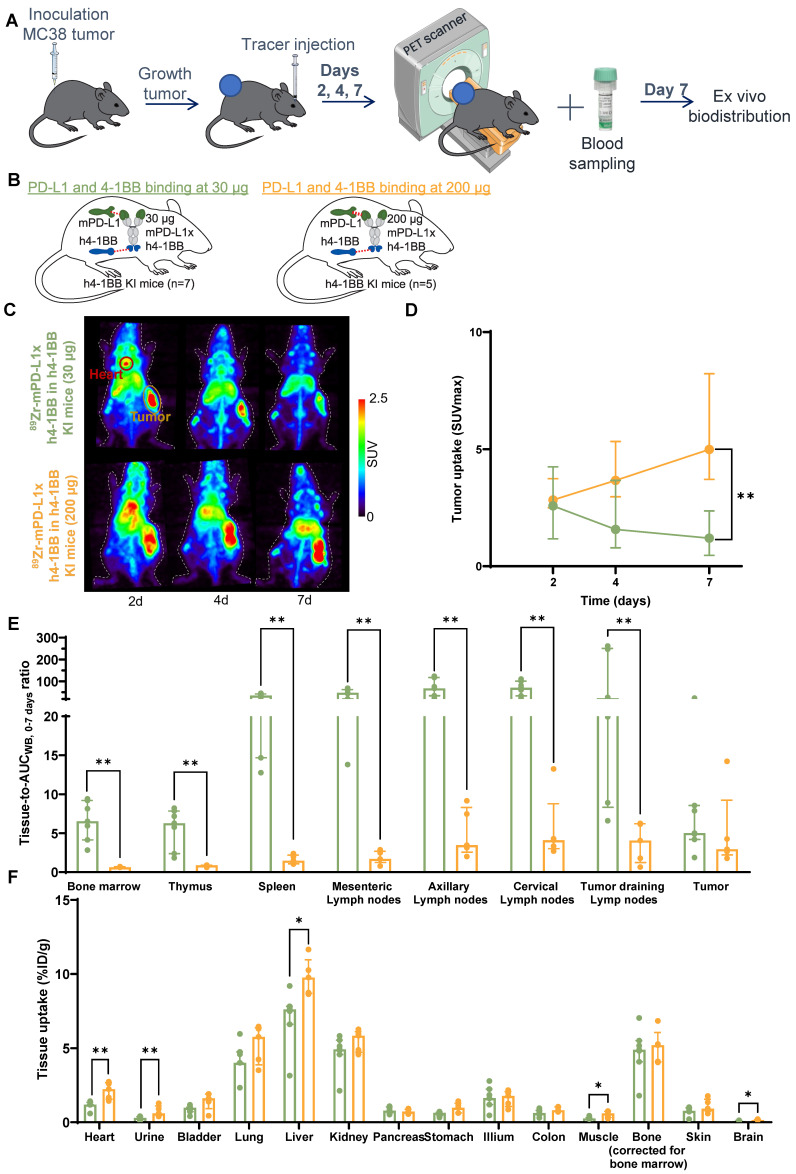
** Dose-dependent biodistribution of ^89^Zr-mPD-L1xh4-1BB in MC38 tumor-bearing h4-1BB KI mice at 30 µg and 200 µg protein dose. (A)** Schematic representation of experimental design. **(B)** Overview of experimental groups and binding specificity of mPD-L1xh4-1BB in h4-1BB KI mice. **(C)** Maximum intensity PET images of MC38 tumor-bearing h4-1BB KI mice that received 30 µg (upper panel) or 200 µg (lower panel) of ^89^Zr-mPD-L1xh4-1BB at 2, 4, and 7 days post-injection. A red circle highlights the heart, and a yellow circle the tumor. **(D)** Tumor uptake in SUVmax at days 2, 4, and 7 post-injection. Statical differences are only presented for day 7 post-injection. **(E)** *Ex vivo* uptake (tissue-to-blood AUC_WB, 0-7 days_) 7 days post-injection in lymphoid tissues and the tumor. **(F)** *Ex vivo* uptake (%ID/g) 7 days post-injection in non-lymphoid tissues. Data are presented as median ± interquartile range. %ID/g: percentage injected dose per gram tissue; AUC_WB, 0-7 days_: whole blood area under the curve from 0 to 7 days; d: day; h: human; h4-1BB KI: human 4-1BB knock-in mice; m: murine; SUV: standard uptake value; *: *P* ≤ 0.05; **: *P* ≤ 0.01.

To further study the dose-dependent biodistribution, two total protein doses of^ 89^Zr-mPD-L1xh4-1BB were compared in MC38 tumor-bearing h4-1BB KI mice: a subtherapeutic dose of 30 µg and a therapeutic dose of 200 µg. In the 30 µg dose group, PET scans showed a slight decrease in tumor uptake from days 2 to 7 post-injection, while the 200 µg dose group showed increasing tumor uptake over time (Figure 4A-D). At day 7 post-injection, a 5.1-fold higher absolute tumor uptake was found for the 200 µg than for the 30 µg dose group (Figure 4D). *Ex vivo* radioactivity analysis revealed comparable tumor-to-AUC_WB, 0-7 days_ ratios for both protein dose groups at day 7 post-injection (Figure 4E). The 30 µg ^89^Zr-mPD-L1xh4-1BB in h4-1BB KI mice showed TMDD with higher tissue-to-AUC_WB, 0-7 days_ ratios compared to the 200 µg dose in all lymphoid tissues, including bone marrow, thymus, spleen, mesenteric, axillary, cervical, and tumor-draining lymph nodes (Figure 4E). The reduced tissue-to-AUC_WB, 0-7 days_ ratios at the 200 µg protein dose suggest partial saturation of targets within the lymphoid tissues as ^89^Zr-Mabcalin competes with unlabeled Mabcalin for target binding. Despite the partial saturation, the uptake in the lymphoid system at a 200 µg dose was still evident. The largest difference in tracer uptake between the dose groups was found in tumor-draining lymph nodes with a 25.2-fold higher tissue-to-AUC_WB, 0-7 days_ ratio for the 30 µg than for the 200 µg protein dose. Thus, the 30 µg protein dose led to prominent distribution toward lymphoid tissues, with peripheral tissue-expressed PD-L1 and 4-1BB acting as the first on-target off-tumor engagement. This resulted in rapid blood clearance and excretion from the body, and therefore modest tumor uptake over time (Figure 3A-D). Given this rapid blood clearance at a 30 µg protein dose, the 200 µg protein dose was selected for the next experiments in which we assessed the target-mediated biodistribution of the PD-L1x4-1BB Mabcalin.

### Target-mediated ^89^Zr-mPD-L1xh4-1BB biodistribution in MC38 tumor-bearing h4-1BB KI mice

**Figure 5:**
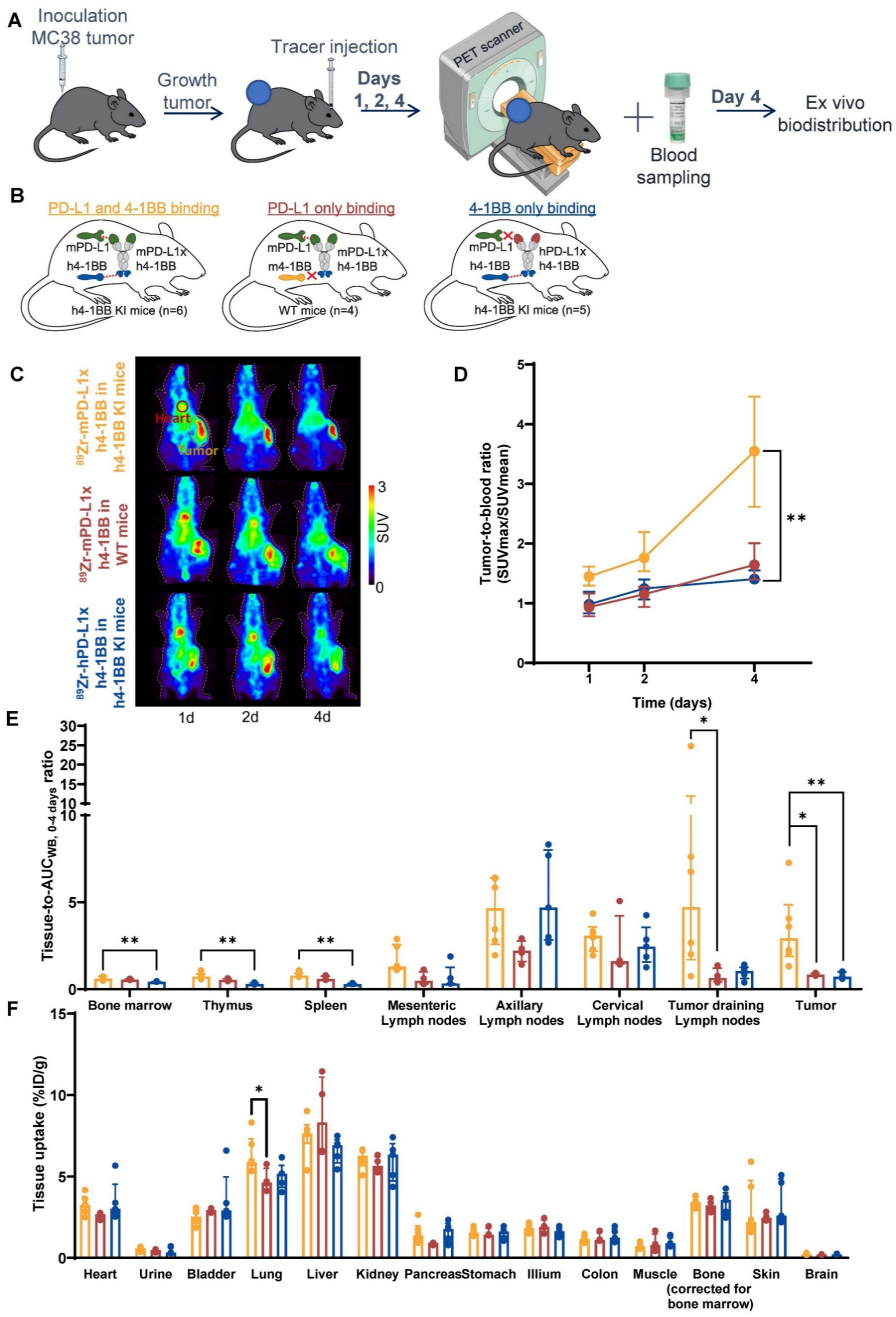
** PD-L1 and 4-1BB target-mediated biodistribution of ^89^Zr-mPD-L1xh4-1BB in MC38 tumor-bearing h4-1BB KI mice. (A)** Schematic representation of experimental design. **(B)** Overview of experimental groups and binding specificity of mPD-L1xh4-1BB and hPD-L1xh4-1BB in h4-1BB KI and WT mice. Both Mabcalins are dosed at 200 µg. **(C)** Maximum intensity PET images of MC38 tumor-bearing h4-1BB KI or WT mice dosed with 200 µg of ^89^Zr-mPD-L1xh4-1BB or ^89^Zr-hPD-L1xh4-1BB at 1, 2, and 4 days post-injection. A red circle highlights the heart, and a yellow circle the tumor. **(D)** *In vivo* uptake in tumor-to-blood ratios at days 1, 2, and 4 post-injection. Statical differences are only presented for day 4, post-injection. **(E)** *Ex vivo* uptake (tissue-to-blood AUC_WB, 0-4 days_ ratio) 4 days post-injection in lymphoid tissues and the tumor. **(F)** *Ex vivo* uptake (%ID/g) 4 days post-injection in non-lymphoid tissues. Data are presented as median ± interquartile range. %ID/g: percentage injected dose per gram tissue; AUC_WB, 0-4 days_: whole blood area under the curve from 0 to 4 days; d: day; h: human; h4-1BB KI mice: human 4-1BB knock-in mice; m: murine; SUV: standard uptake value; WT mice: wild-type mice; *: *P* ≤ 0.05; **: *P* ≤ 0.01.

To evaluate target-mediated biodistribution mediated by PD-L1 and 4-1BB, ^89^Zr-mPD-L1xh4-1BB or ^89^Zr-hPD-L1xh4-1BB were administered to MC38 tumor-bearing h4-1BB KI and WT mice. This allowed us to compare dual PD-L1 and 4-1BB binding with PD-L1 only and 4-1BB only binding (Figure 5B). Imaging up to 4 days was preferred to minimize the effect of a difference in tumor growth attributable to the therapeutic effect of the 200 µg ^89^Zr-mPD-L1xh4-1BB dose in h4-1BB KI mice (Figure S8A). The ^89^Zr-mPD-L1xh4-1BB in h4-1BB KI mice, binding both its targets, showed higher tumor uptake than the ^89^Zr-mPD-L1xh4-1BB in WT mice, lacking human 4-1BB, and binding only murine PD-L1. PET imaging revealed higher tumor-to-blood ratios for ^89^Zr-mPD-L1xh4-1BB in h4-1BB KI mice than in WT mice. Specifically, the ratios were 1.5-fold (*P* ≤ 0.01), 1.7-fold (*P* = ns), and 2.1-fold (*P* = ns) higher on days 1, 2, and 4, respectively (Figure 5A-D). Similarly, in h4-1BB KI mice, we found higher tumor-to-blood ratios for ^89^Zr-mPD-L1xh4-1BB than for ^89^Zr-hPD-L1xh4-1BB, namely 1.4-fold (*P* ≤ 0.05), 1.5-fold (*P* ≤ 0.05), and 2.5-fold (*P* ≤ 0.01) higher on respectively days 1, 2, and 4 (Figure 5C, D). Thus, ^89^Zr-mPD-L1xh4-1BB, able to bind PD-L1 and 4-1BB in h4-1BB KI mice, showed higher tumor uptake than ^89^Zr-hPD-L1xh4-1BB, which binds only 4-1BB in h4-1BB KI mice.

*Ex vivo* radioactivity analysis at day 4 confirmed these observations with respectively 4.1-fold and 4.3-fold higher tumor-to-AUC_WB, 0-4 days_ ratios for ^89^Zr-mPD-L1xh4-1BB in h4-1BB KI mice than for the ^89^Zr-PD-L1x4-1BB Mabcalins, which bind either PD-L1 or 4-1BB in their respective mouse model (Figure 5E). Bone marrow, thymus, and spleen *ex vivo* tissue-to-AUC_WB, 0-4 days_ ratios, in h4-1BB KI mice, were higher for ^89^Zr-mPD-L1xh4-1BB than for ^89^Zr-hPD-L1xh4-1BB. However, similar bone marrow, thymus, and spleen tissue-to-AUC_WB, 0-4 days_ ratios were found for the ^89^Zr-mPD-L1xh4-1BB in WT mice, indicating that uptake in these lymphoid organs was more correlated with PD-L1 than 4-1BB-binding. ^89^Zr-mPD-L1xh4-1BB tumor-draining lymph node uptake was higher in h4-1BB KI mice than in WT mice, suggesting a role for 4-1BB (Figure 5E). Tracer uptake in normal non-lymphoid tissues was similar for ^89^Zr-mPD-L1xh4-1BB in h4-1BB KI and WT mice and ^89^Zr-hPD-L1xh4-1BB in h4-1BB KI mice (Figure 5F). This was also the case for the liver uptake despite the well-known 4-1BB expression there. Overall, at the 200 µg protein dose, dual PD-L1 and 4-1BB engagement is associated with the tumor ^89^Zr-PD-L1x4-1BB Mabcalin uptake, while bone marrow, thymus, and spleen uptake were primarily mediated by PD-L1 than 4-1BB binding.

### PD-L1, CD3, CD8, and 4-1BB tumor protein expression in MC38 tumor-bearing mice exposed to PD-L1x4-1BB Mabcalins

**Figure 6:**
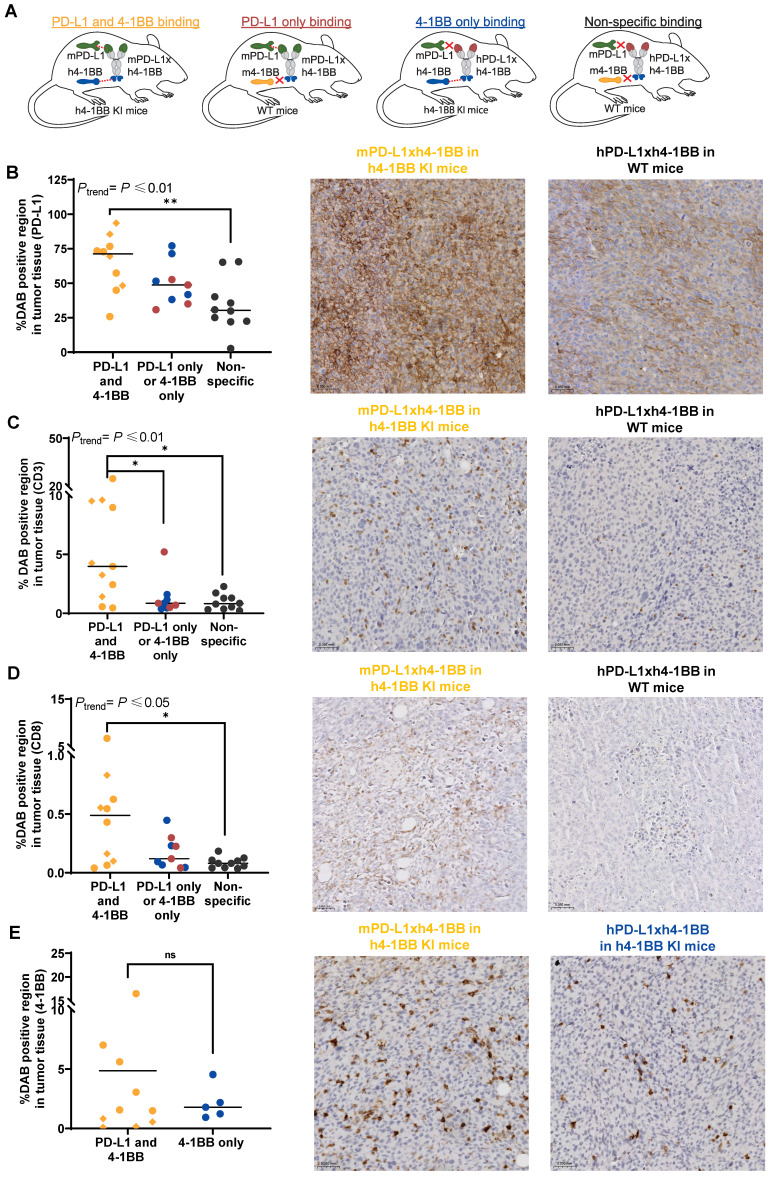
** PD-L1, 4-1BB, CD3, and CD8 tumor expression in MC38 tumor-bearing h4-1BB KI or WT mice that received ^89^Zr-mPD-L1xh4-1BB or ^89^Zr-hPD-L1xh4-1BB. (A)** Overview of experimental groups and binding specificity of mPD-L1xh4-1BB and hPD-L1xh4-1BB in h4-1BB KI and WT mice. **(B)** Quantification of PD-L1 expression **(C)** Quantification of CD3 expression **(D)** Quantification of CD8 expression **(E)** Quantification of 4-1BB expression. Representative IHC images are depicted horizontally next to the quantification graphs. The quantifications are expressed as a percentage of DAB-positive regions in tumor tissue. Tumor expression levels of the PD-L1 and 4-1BB binding group at 200 µg 4 days post-injection are presented as yellow dots and 7 days post-injection as yellow diamonds. Tumor expression levels at day 4 post-injection in the PD-L1 binding group are presented as red dots and the 4-1BB binding group as blue dots, both are dosed at 200 µg. In the non-specific binding group, tumor expression levels of the 30 µg dose at 7 days post-injection and 200 µg at 4 days post-injection are presented. DAB: diaminobenzidine; h: human; IHC: immunohistochemistry; m: murine; ns: not significant; *: *P* ≤ 0.05; **: *P* ≤ 0.01.

To interpret our *in vivo* biodistribution findings, we immunohistochemically evaluated PD-L1, 4-1BB, CD3, and CD8 expression in MC38 tumors collected from h4-1BB KI or WT mice that received mPD-L1xh4-1BB or hPD-L1xh4-1BB (Figure 6A). PD-L1 was expected to be expressed on tumor and immune cells, CD3 on T-cells, CD8 on activated T-cells, and 4-1BB on immune cells. PD-L1 expression was 1.9-fold higher in tumors of h4-1BB KI mice dosed with 200 µg mPD-L1xh4-1BB than in tumors of WT mice dosed with control hPD-L1xh4-1BB. This suggests PD-L1 upregulation due to dual PD-L1 and 4-1BB engagement compared to the non-specific binding control lacking the possibility of PD-L1 and 4-1BB binding (*P* ≤ 0.01; Figure 6B).

We observed a trend with the highest PD-L1 protein expression in tumors of h4-1BB KI mice that received 200 µg mPD-L1xh4-1BB, followed by WT mice given 200 µg mPD-L1xh4-1BB, and h4-1BB KI mice dosed with 200 µg hPD-L1xh4-1BB. The lowest expression occurred in the control tumors of WT mice that received 30 µg or 200 µg of the non-specific control hPD-L1xh4-1BB (*P*_trend_ ≤ 0.01; Figure 6B). Similar trends as for PD-L1 were observed for CD3 (*P*_trend_ ≤ 0.01) and CD8 protein (*P*_trend_ ≤ 0.05) expression in the tumor (Figure 6C-D). Tumors collected from h4-1BB KI mice dosed with 200 µg of mPD-L1xh4-1BB had a 6.5-fold higher CD3 and 11.8-fold higher CD8 protein expression (both *P* ≤ 0.05) than observed in tumors from WT mice given 30 µg or 200 µg hPD-L1xh4-1BB. This indicates T-cell infiltration and/or proliferation and activation induced by mPD-L1xh4-1BB in tumors of h4-1BB KI mice (Figure 6C-D). Human 4-1BB protein expression was only stained in tumors of h4-1BB KI mice. Its expression did not differ between tumors in h4-1BB KI mice that received mPD-L1xh4-1BB or hPD-L1xh4-1BB. This suggests that 4-1BB expression within the tumor was either not induced or not detected (Figure 6E). Taken together, the PD-L1, CD3, and CD8 expression in the tumor was increased more in the 200 µg mPD-L1xh4-1BB dosed h4-1BB KI mice than in mPD-L1xh4-1BB dosed WT mice and hPD-L1xh4-1BB dosed h4-1BB KI mice.

### Intratumoral distribution of ^89^Zr-labeled PD-L1x4-1BB Mabcalins

**Figure 7:**
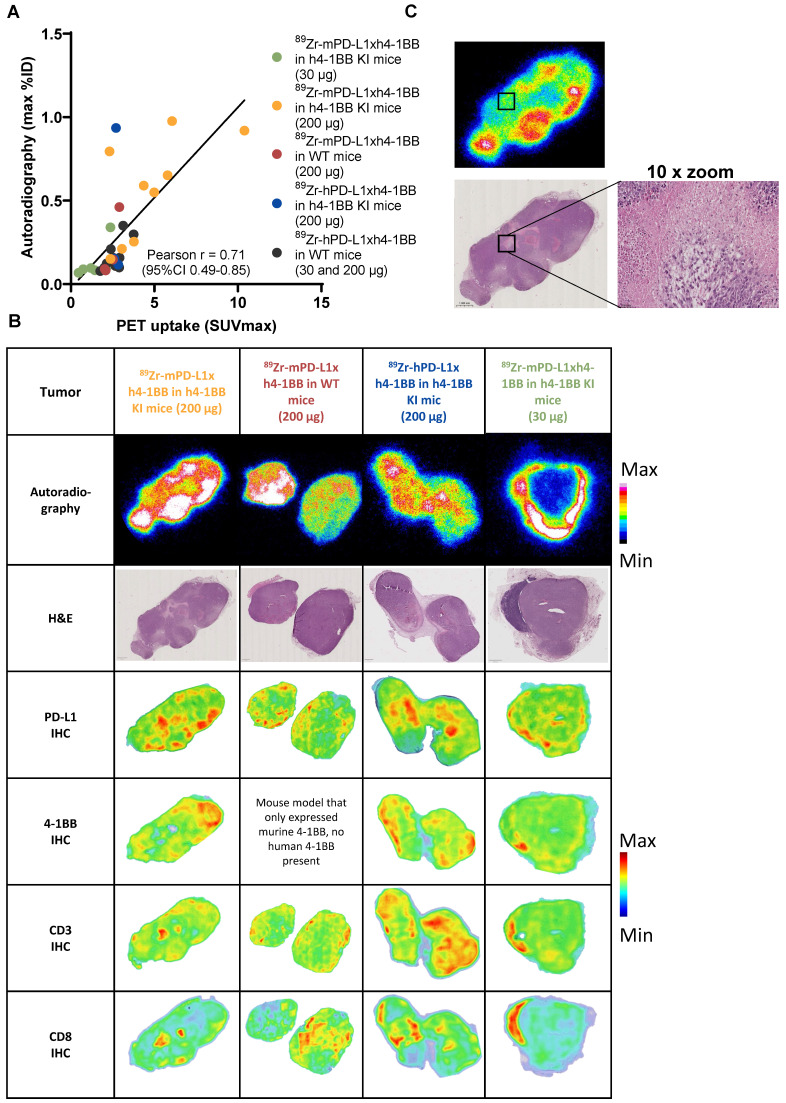
** Intratumoral tumor distribution of the autoradiography signal of ^89^Zr-mPD-L1xh4-1BB and ^89^Zr-hPD-L1xh4-1BB in relation to PD-L1, 4-1BB, CD3, and CD8 protein expression. (A)** Correlation between PET uptake and autoradiography signal in a 4 µm tumor section. **(B)** Autoradiography images of tumors of h4-1BB KI or WT mice given 30 µg or 200 µg ^89^Zr-mPD-L1xh4-1BB and ^89^Zr-hPD-L1xh4-1BB, followed by H&E staining of the same section. Density maps of PD-L1, 4-1BB, CD3, and CD8 IHC stained consecutive sections. **(C)** Autoradiography and H&E images of tumor of h4-1BB KI mice given 200 µg ^89^Zr-mPD-L1xh4-1BB 4 days, post-injection. A necrotic area is indicated with a box and a 10x magnification image. Representative data are shown. %ID: percentage injected dose; h: human; h4-1BB KI mice: human 4-1BB knock-in mice; H&E: hematoxylin and eosin; IHC: Immunohistochemistry; m: murine; WT mice: wild-type mice.

To study the intratumoral distribution of ^89^Zr-PD-L1x4-1BB Mabcalins at two doses, autoradiography was performed on tumor tissue sections. The autoradiography signal correlated with SUVmax of the individual tumors (Figure 7A). The intratumoral radioactivity signal of the ^89^Zr-PD-L1x4-1BB Mabcalins was distributed heterogeneously in all groups with localized radioactivity hotspots and areas with low signal (Figure 7B). The ^89^Zr-mPD-L1xh4-1BB signal in tumors of h4-1BB KI mice dosed at 30 µg was predominantly localized at the rim of the tumor, suggesting limited tumor penetration. At the 200 µg protein dose, this ^89^Zr-mPD-L1xh4-1BB signal was more homogeneous in all tumors of h4-1BB KI mice and also detected in the tumor's center. This autoradiography signal did not colocalize with necrotic or apoptotic areas (Figure 7C). Furthermore, the ^89^Zr-mPD-L1xh4-1BB signal in tumors of h4-1BB KI mice mainly colocalized with PD-L1 expression at doses of 30 µg and 200 µg, suggesting that intratumoral uptake and distribution of ^89^Zr-mPD-L1xh4-1BB in h4-1BB KI is likely associated with PD-L1 expression. Additionally, in the tumors of WT mice that received ^89^Zr-mPD-L1xh4-1BB, some areas in the tumor section showed colocalization between PD-L1 expression and ^89^Zr-mPD-L1xh4-1BB uptake, indicating specific PD-L1-mediated uptake in these regions. Similarly, ^89^Zr-hPD-L1xh4-1BB signal in tumors of h4-1BB KI mice colocalized with 4-1BB expression in some areas. Moreover, CD3 expression colocalized with 4-1BB and CD8 expression in all groups, suggesting that 4-1BB and CD8 are co-expressed on T-cells in the tumor (Figure 7B).

## Discussion

This study revealed high, specific, and PD-L1 mediated ^89^Zr-PD-L1x4-1BB uptake in the bone marrow, thymus, and spleen. At a low protein dose, this target-mediated drug disposition resulted in fast drug clearance from the blood. Moreover, engaging both PD-L1 and 4-1BB, rather than only PD-L1 or only 4-1BB, increased the PD-L1, CD3, and CD8 expression in the tumor and tumor uptake of the bispecific antibody.

This is the first study, using molecular imaging, to illustrate that an increase in target expression within the tumor - triggered by dual PD-L1 and 4-1BB engagement of the bispecific antibody, in contrast to single target engagement - correlates with increased tumor uptake of the radiolabeled PD-L1x4-1BB bispecific antibody 20. The anticipated mechanism of PD-L1 upregulation by PD-L1x4-1BB bispecific antibodies likely involves 4-1BB engagement, which stimulates T-cells to produce IL-2, IL-4, and interferon-gamma (IFN-γ) 21. Subsequently, IFN-γ triggers the inducible PD-L1 expression of the tumor's cancer and/or immune cells 22. A previous study showed that intratumoral INF-γ injections increased the ^99m^Tc-labeled anti-PD-L1 antibody tumor uptake in MC38 tumor-bearing mice 23. In our study, the increase in CD3 and CD8-expressing cells in the tumor suggests a productive Mabcalin activity by mounting an effective immune response. This immune response is expected to drive the increase of PD-L1 expression on tumor and/or immune cells. Most likely, the elevated PD-L1 expression induced by the PD-L1x4-1BB Mabcalin is associated with the ^89^Zr-PD-L1x4-1BB tumor accumulation as its intratumoral radioactivity signal colocalized with PD-L1 and not with 4-1BB or T-cell distribution.

The target-mediated biodistribution of immunomodulatory bispecific antibodies is not fully elucidated due to a lack of *in vivo* models that allow the study of fully human-reactive antibodies. The current study, using two Mabcalin molecules and two tumor-bearing mice models, provided a unique setting using PET imaging and *ex vivo* analysis to unveil the PD-L1x4-1BB Mabcalin's biodistribution and the influence of dual and single target engagement on the biodistribution. A recent study with another tetravalent anti-murine/human PD-L1 and anti-human 4-1BB antibody demonstrated PD-L1-mediated biodistribution in human PD-L1-positive tumor-bearing immunodeficient mice, with also high uptake in the tumor and spleen 24. However, the model used lacked human 4-1BB expression, thus neglecting the influence of 4-1BB and dual PD-L1 and 4-1BB binding on the biodistribution. A study with a ^125^I-labeled trispecific scMATCH3, targeting human PD-L1, 4-1BB, and serum albumin showed tumor retention that peaked at 24 h in immunodeficient BALB/c nude mice bearing human PD-L1-positive tumors 25. Here, the mice model also lacked human 4-1BB and human PD-L1 in non-tumor tissues and, therefore, only allowed the analysis of PD-L1-mediated distribution in the tumor. This absence of peripheral PD-L1 and 4-1BB is especially important in the context of T-cell immunostimulating antibodies, as illustrated for antibodies targeting the PD-1/PD-L1 pathway, with activity in lymph nodes being critical to mount a durable T-cell response that is ultimately associated with tumor eradication 26.

A limitation of the h4-1BB KI mice is the absence of a humanized 4-1BB ligand, which may leave human 4-1BB receptors largely unoccupied. As a result, more 4-1BB receptors may be available for binding than in a physiological setting where the ligand is present and actively engaging the receptor. This could potentially lead to increased Mabcalin tissue uptake due to 4-1BB binding compared to the real-world scenario. The specificity of mPD-L1xh4-1BB in h4-1BB was confirmed by comparing its biodistribution to the non-binding control hPD-L1xh4-1BB in WT mice. In conventional tracer studies, specificity is often demonstrated using an unlabeled compound to block the uptake of the radiolabeled compound. In this study, it would require administering a high dose of mPD-L1xh4-1BB to block the ^89^Zr-mPD-L1xh4-1BB signal in h4-1BB mice. However, mPD-L1xh4-1BB Mabcalin in h4-1BB KI mice is a biologically active therapeutic agent, capable of eliciting a pharmacodynamic response in h4-1BB KI mice. Administering high doses of this Mabcalin can result in severe toxicities and may even cause complete tumor regression in these mice, making it impossible to quantify tracer uptake. In these cases, the tumor may be too small to quantify with PET imaging, or it may have been entirely eliminated. Moreover, the mPD-L1xh4-1BB Mabcalin in h4-1BB KI mice upregulated PD-L1 tumor target expression, which further complicates or even precludes complete blocking of tumor uptake. This upregulation increases the availability of PD-L1 binding sites, making it difficult to saturate all targets with the blocking dose. As a result, it becomes challenging to distinguish between specific and non-specific tracer signals, since residual uptake may reflect either incomplete blockade of PD-L1 or non-specific uptake.

To study the target-mediated biodistribution of the PD-L1x4-1BB Mabcalin, we employed a novel approach using two Mabcalins and two mouse models. This strategy was chosen over conventional single-target tracer controls, as single-binding Mabcalins were unavailable. Alternative design controls lack the specific Mabcalin molecular format, which prevents appropriate comparability. Also, our design, using mPD-L1xh4-1BB in WT mice and hPD-L1xh4-1BB in h4-1BB KI mice, can be interpreted as a single-target tracer control. In both conditions, one binding arm is specific to PD-L1 or 4-1BB, while the other binding arm binds non-specifically due to the absence of its target in the mouse model. Moreover, an alternative design could involve the use of a 4-1BB-blocking antibody. However, it is important to note that 4-1BB is a biologically active receptor capable of inducing anti-tumor responses and upregulating PD-L1 expression. Introducing a blocking antibody in this context would add an additional layer of biological complexity, complicating the interpretation of biodistribution data. In conclusion, we consider the dual-model and construct approach to provide a robust and interpretable framework to distinguish the individual contributions of PD-L1 and 4-1BB binding *in vivo*.

We tested two tracer doses and selected the 200 µg dose to minimize excessive TMDD of mPD-L1xh4-1BB in h4-1BB mice. The dose may not have been optimal to study the biodistribution of mPD-L1xh4-1BB in WT mice or hPD-L1xh4-1BB in h4-1BB KI mice, as receptor saturation could have influenced the biodistribution profile. Also, the 200 µg dose of^ 89^Zr-mPD-L1xh4-1BB in resulted in tumor shrinkage and cell death in h4-BB KI. . Potentially, this might impact the tumor tracer uptake because the read-out is expressed as a percentage injected dose per gram tissue. However, on day 2, the tumor uptake was already higher in the group where both PD-L1 and 4-1BB could be bound compared to the group binding to either PD-L1 or 4-1BB, while the tumor sizes were still similar. Thus, the increased tumor uptake, at least on day 2, is irrespective of tumor shrinkage. We and others demonstrated infiltration and activation of lymphocytes in the tumors of PD-L1-positive tumor-bearing mice due to bispecific PD-L1x4-1BB antibody therapy 6, 27-31. Since 4-1BB is primarily expressed on activated cytotoxic CD8-positive T-cells and helper CD4-positive T-cells, T-cell infiltration and activation consequently likely lead to increased 4-1BB expression within the tumor microenvironment 4. Single-cell transcriptomic analysis of tumor-infiltrated lymphocytes in MC38 tumor-bearing h4-1BB KI mice showed that this was the case for another tetravalent PD-L1x4-1BB bispecific antibody, which induced 4-1BB expression on T-cells 21*.* Our IHC tumor tissue analysis did not show an increase in 4-1BB expression after administering 200 µg mPD-L1xh4-1BB to PD-L1-positive murine tumor-bearing h4-1BB KI mice. Possibly, earlier transient expression of 4-1BB on activated T-cells already occurred and had ceased by the time of tumor collection 31. Alternatively, the group size might be insufficient to detect a difference in 4-1BB expression. Lastly, it is possible that the IHC analysis, which was performed on a single tumor section, does not fully capture the spatial heterogeneity of 4-1BB expression throughout the entire tumor.

The intratumoral expression of CD3, CD8, and 4-1BB was heterogeneous. This reflects the expected biological distribution of immune cells within the tumor microenvironment. These markers are expressed on infiltrating immune cells, and their localization is influenced by factors such as immune cell infiltration patterns and the presence of tumor-associated lymphoid structures, which are inherently variable across and within tumors. Although the tumor cell line used in this study is PD-L1-positive, we observed notable heterogeneity in PD-L1 expression within the tumor tissue. This variability likely reflects the dynamic regulation of PD-L1 by the tumor microenvironment, including factors such as local cytokine signaling 32. Additionally, PD-L1 expression is not limited to tumor cells but can also be found on tumor-associated macrophages and other immune cells, further contributing to the heterogeneous staining patterns observed 9. The extent of antibody internalization is crucial for interpreting tissue radioactivity uptake. When a ^89^Zr-radiolabeled antibody internalizes, ^89^Zr can residualize and accumulate. To enable quantitative assessment of internalization in tumor and immune cells, we developed a flow cytometry-based method that overcomes limitations of conventional techniques, like stripping, which are unsuited for immune cells and microscopy that lacks quantitative read-out. Using immune cell populations from three distinct donors introduced greater biological variability in target expression compared to the tumor cells, which in turn led to increased variation in the internalization data. We showed PD-L1x4-1BB Mabcalin internalization in stimulated 4-1BB expressing T-cells and also in PD-L1-positive tumor cells. In tumor cells but not in T-cells, we found PD-L1 receptor recycling to the cell membrane after internalization. Rapid receptor recycling was also described for monospecific anti-PD-L1 antibodies 33-35. *In vivo*, we observed ^89^Zr accumulation over time in tumors of h4-1BB KI mice dosed with 200 µg ^89^Zr-mPD-L1x4-1BB, also indicating continuous binding and internalization.

Agonistic 4-1BB antibodies, like urelumab, have encountered a significant challenge of on-target off-tumor liver toxicity in clinical trials, leading to the activation of Kupffer cells in the liver and hepatocyte damage 4. It has been reported that a high-affinity difference between cross-linking targets (e.g., tumor antigens or PD-L1) and 4-1BB leads to potential functional advantages of the bispecific molecules over monospecific agonist 4-1BB antibodies. Such an advantage has been shown with the trispecific scMATCH3 targeting human PD-L1, 4-1BB, and human serum albumin that was designed with a high affinity ratio difference between PD-L1 and 4-1BB to maximize 4-1BB activation and improve the therapeutic window *in vitro* 25. In this context, another PD-L1x4-1BB tetravalent bispecific antibody was designed with even a ~65-fold higher affinity for PD-L1 than 4-1BB, aiming to overcome on-target hepatoxicity 21. Our bispecific PD-L1x4-1BB Mabcalin is designed to distribute mainly to PD-L1-positive tissues with a seven-fold higher affinity for PD-L1 than for 4-1BB. We did not observe differences in *ex vivo* liver radioactivity uptake between groups with or without 4-1BB binding, suggesting that PD-L1x4-1BB Mabcalin liver uptake is not mediated by 4-1BB.

We observed TMDD of mPD-L1×h4-1BB in h4-1BB knock-in mice, demonstrating that target occupancy is dose-dependent. For bispecific antibodies, which require simultaneous engagement of both target antigens for their therapeutic effect, quantifying target occupancy is particularly important. Mechanistic modeling with PET and pharmacokinetic data offers an approach to estimate target occupancy across different dose levels. This approach uses a compartmental model to represent how radiopharmaceuticals distribute within the body, with each compartment corresponding to a specific tissue or biological system. It can also capture key biological processes within compartments, such as internalization, binding, and clearance. The model structure can be tailored to the specific radiopharmaceutical but typically includes at least the central (blood) compartment and a tumor compartment, enabling the distinction between free, bound, and internalized antibodies 36-38. Once the model is established, it can be used to simulate different dosing scenarios and predict the resulting levels of receptor occupancy.

These preclinical biodistribution data combined with *ex vivo* tumor tissue analysis and internalization data in relevant models can further guide the development of PD-L1 and 4-1BB targeting bispecifics.

## Supplementary materials

Supplementary figures and tables.

## Data Availability

The raw data generated and described in this article are available in the supplemental data. Other data are available from the corresponding author (m.n.de.hooge@umcg.nl) upon reasonable request.

## Abbreviations

%ID: percentage injected dose
4-1BB: CD137
^89^Zr: zirconium-89
AUC: area under the time activity curve
AUC_WB_: area under the time activity curve in whole blood
AF647: Alexa Fluor 647^TM^
CD3: cluster of differentiation 3
DAB: diaminobenzidine chromogen
g: gram
h4-1BB KI: human 4-1BB knock-in mice
HER2: epidermal growth factor receptor 2
hPD-L1xh4-1BB: Mabcalin targeting human PD-L1 and human 4-1BB
IFN-γ: interferon-gamma
IHC: immunohistochemistry
IL-2: interleukin 2
Mabcalin: antibody-anticalin fusion protein
MC38: murine colon adenocarcinoma
MFI: mean fluorescence intensity
mPD-L1xh4-1BB: Mabcalin targeting murine PD-L1 and human 4-1BB
PBMC: peripheral blood mononuclear cell
PD-1: programmed cell death protein 1
PD-L1: programmed cell death ligand 1
PD-L1 KO ES2: PD-L1 knock-out ES2 cells
PET: positron emission tomography
PMA: phorbol 12-myristate 13-acetate
PBS: phosphate-buffered saline
SUVmax: maximum standard uptake value
SUVmean: mean standard uptake value
SPECT: single-photon emission computed tomography
TMDD: target-mediated drug disposition
WT: wild-type mice
