# Supplementary material for: Elevated target expression by dual PD-L1 and 4-1BB engagement is associated with 89Zr-PD-L1x4-1BB bispecific Mabcalin tumor uptake
Source: Theranostics. 2026 Apr 22;16(11):6184–201. doi: 10.7150/thno.123930 (PMC13142236; doi:10.7150/thno.123930)
Supplement: Supplementary file 1 — Supplementary figures and tables. [file thnov16p6184s1.pdf]

Supplementary data for

Elevated target expression by dual PD-L1 and 4-1BB engagement is associated with ^{89}Zr-PD-L1x4-1BB bispecific Mabcalin tumor uptake

Claudia A.J. van Winkel, Xiaoyu Fan, Danique Giesen, Glenn Gauderat, Steven de Jong, Thomas Jaquin, Wim Timens, Agathe Lepissier, Marleen Richter, Stefan Grüner, Nicole Andersen, Laurent Laboureur, Lucia Pattarini, Helene Lelièvre, Elisabeth G.E. de Vries, Aizea Morales-Kastresana, Marjolijn N. Lub-de Hooge*

*Corresponding author. Email: m.n.de.hooge@umcg.nl

The file includes:

Supplemental Methods
Supplemental References
Figure S1. Characterization of mPD-L1xh4-1BB and hPD-L1xh4-1BB.
Figure S2. Characterization of biotinylated hPD-L1xh4-1BB.
Figure S3. Characterization of DFO-conjugated and ^{89}Zr-radiolabeled mPD-L1xh4-1BB and hPD-L1xh4-1BB.
Figure S4. PD-L1 and 4-1BB expression of ionomycin + PMA or IL-2 + PHA stimulated human T-cells.
Figure S5. Flow cytometry gating strategy used in internalization experiments.
Figure S6. Specificity of ^{89}Zr-mPD-L1xh4-1BB in MC38-bearing h4-1BB KI mice dosed at 30 µg.
Figure S7. Specificity of ^{89}Zr-mPD-L1xh4-1BB in MC38-bearing h4-1BB KI mice dosed at 200 µg.
Figure S8. Tumor size during the imaging time frame.

Table S1. In vivo tumor uptake (SUVmax) and in vivo tumor-to-ex vivo blood ratio (SUVmax/SUVmean) of ^{89}Zr-mPD-L1x4-1BB and ^{89}Zr-hPD-L1x4-1BB in h4-1BB KI or WT mice at 2, 4 and 7 days post-injection.
Table S2. In vivo tumor-to-blood ratio (SUVmax/SUVmean) of ^{89}Zr-mPD-L1x4-1BB and ^{89}Zr-hPD-L1x4-1BB in h4-1BB KI or WT mice at 1, 2 and 4 days post-injection.
Table S3. Absolute ex vivo uptake (%ID/g) of ^{89}Zr-mPD-L1x4-1BB and ^{89}Zr-hPD-L1x4-1BB in h4-1BB KI or WT mice at 7 days post-injection in lymphoid tissues and the tumor.
Table S4. Ex vivo uptake (tissue-to-blood $AUC_{WB,\ 0\text{-}7\ days}$) of ^{89}Zr-mPD-L1x4-1BB and ^{89}Zr-hPD-L1x4-1BB in h4-1BB KI or WT mice at 7 days post-injection in lymphoid tissues and the tumor.
Table S5. Absolute ex vivo uptake (%ID/g) of ^{89}Zr-mPD-L1x4-1BB and ^{89}Zr-hPD-L1x4-1BB in h4-1BB KI or WT mice at 4 days post-injection in lymphoid tissues and the tumor.

Table S6. Ex vivo uptake (tissue-to-blood $AUC_{WB,\ 0\text{-}4\ days}$) of ^{89}Zr-mPD-L1x4-1BB and ^{89}Zr-hPD-L1x4-1BB in h4-1BB KI or WT mice at 4 days post-injection in lymphoid tissues and the tumor.
Table S7. Ex vivo uptake (tissue-to-blood $AUC_{WB,\ 0\text{-}7\ days}$) of ^{89}Zr-mPD-L1x4-1BB and ^{89}Zr-hPD-L1x4-1BB in h4-1BB KI or WT mice at 7 days post-injection in non-lymphoid tissues.
Table S8. Ex vivo uptake (tissue-to-blood $AUC_{WB,\ 0\text{-}7\ days}$) of ^{89}Zr-mPD-L1x4-1BB and ^{89}Zr-hPD-L1x4-1BB in h4-1BB KI or WT mice at 4 days post-injection in non-lymphoid tissues.

Supplemental Methods

Conjugation and ^{89}Zr-labeling of mPD-L1xh4-1BB and hPD-L1xh4-1BB

mPD-L1xh4-1BB and hPD-L1xh4-1BB Mabcalins (Servier and Pieris Pharmaceuticals) were conjugated after a buffer exchange to 25 mM $NaHCO_3$ in 0.9% NaCl pH 9.0 with a PD-10 desalting column (Cytiva, #28918007), followed by 60 min incubation with p-SCN-Bn-deferoxamine (DFO; Macrocyclis, #B-705) at 20 °C. The DFO-conjugated Mabcalins were purified using a PD-10 desalting column and formulated in 50 mM histidine (Sigma-Aldrich), 50 mM NaCl (Sigma-Aldrich), 200 mM arginine (Sigma-Aldrich), 0.01% polysorbate 80 (Fisher Scientific), pH 6.5 and stored at -80 °C DFO-PD-L1x4-1BB Mabcalins were radiolabeled with ^{89}Zr, as described previously [1, 2]. Potential aggregation and fragmentation of the conjugated and radiolabeled PD-L1x4-1BB were assessed with size exclusion ultra-high performance liquid chromatography equipped with BioSep or Yarra gel filtration SEC-s3000 column (Phenomenex, # 00H-2146-K0, # 00H-4513-K0), as previously described (Figure S3C-F) [3]. Radiochemical purity was measured by trichloroacetic acid precipitation assay [4].

The potential targets for DFO-conjugation to surface-exposed lysine side chains of the hPD-L1xh4-1BB were determined by homology modeling with Molecular Operating Environment (MOE) 2020.09 (Chemical Computing Group ULC, Montreal, QC, Canada). The Anticalin protein moiety targeting 4-1BB was homology modeled based on the protein data bank templates 1DFV. The fragment antigen binding region of the hPD-L1 antibody building block was modeled using the antibody modeler function of MOE with the following template structures for the variable light chain: PDB ID.chain for frameworks, CDRs 1, 2, 3: 4R7D.H, 1YY9.C, 4YXL.L, 3HFM.L, and for the variable heavy chain: 4R7D.G, 2GK0.H,

2GK0.H, 1SBS.H. This modeling revealed two surface-exposed lysine residues close to the complementarity-determining regions targeting PD-L1 and seven surface-exposed lysine residues in the 4-1BB targeting binding regions of the Anticalin portion, respectively (Figure S3A-B). Therefore, multiple DFO to Mabcalin ratios were investigated to determine the effect of DFO-conjugation on binding affinities.

To evaluate the binding properties of DFO-PD-L1x4-1BB for 4-1BB and PD-L1, the relative potency, expressed as half-effective concentration (EC50) of DFO-Mabcalin divided by EC50 of their respective unconjugated Mabcalin, was determined with an indirect enzyme-linked immunosorbent assay (ELISA) as described before [5]. The PD-L1 and 4-1BB extracellular domains (R&D Sytems, #9049-B7-100, #9220-4B-100) were used as a target. DFO-conjugation did not affect PD-L1 binding, while 4-1BB binding was already impaired at a 1:2 ratio of PD-L1x4-1BB Mabcalin to DFO. Therefore, the lowest possible Mabcalin to DFO ratio of 1:2 needed for ^{89}Zr labeling at 30 – 167 MBq/mg with ≥ 95% radiochemical purity was considered optimal (Figure S3C-L). ^{89}Zr radiolabeling of DFO-hPD-L1xh4-1BB did not affect target binding based on a radioactive ELISA (Figure S3K, L) [6]. DFO-hPD-L1xh4-1BB can activate T-cells to the same degree as unmodified Mabcalin based on a staphylococcal enterotoxin B functional assay (Figure S3M) [7].

Pharmacokinetics of DFO-hPD-L1xh4-1BB in CD1 mice

The effect of DFO-conjugation on the PK profile of hPD-L1xh4-1BB was tested in male CD1 mice (Charles River Laboratories). The National Committee for Ethical Reflection on Animal Experimentation in France approved the study. hPD-L1xh4-1BB or DFO-hPD-L1xh4-1BB 10 mg/mL was administered intravenously in the caudal vein (n = 6 mice per group, 9 weeks of age). The mice were divided into two groups (n = 3 per time point), and micro-blood sampling was performed at 5 min, 4, 24, and 96 h or 1, 8, 48, and

168 h. The (DFO-)hPD-L1xh4-1BB plasma concentrations were determined with a MesoScale Discovery assay. A 384-well multi-array plate was coated overnight at 4°C with hPD-L1 at 1 µg/mL. After blocking with 2% bovine serum albumin and 0.1% polysorbate 20 in PBS (Gibco, pH 7.4), the collected plasma was incubated for 1 h at room temperature. (DFO-)hPD-L1xh4-1BB was detected with 1 µg/mL anti-NGAL Ab (clone PL713, BioGenes), anti-rabbit IgG Sulfo-Tag (Meso Scale Discovery; #R32AB-1), and 2x MSD Read Buffer (Meso Scale Discovery, #R92TC-1). The plates were measured using Sector Imager S600. The area under the plasma concentration-time curve over 168 h, half-life, and clearance were calculated with non-compartmental PK analysis with an in-house macro in Microsoft Excel software version office 365 (Servier; Figure S3N, O).

Plasma tracer integrity and autoradiography of tissues

Plasma tracer integrity of ^{89}Zr-mPD-L1xh4-1BB and ^{89}Zr-hPD-L1xh4-1BB were confirmed with SDS-PAGE (Figure 3E). A 10% polyacrylamide gel (Bio-Rad; #4561034) was loaded with 250 counts/min of the plasma samples. The positive control, the injected tracer, stored at 4°C in 0.5% human serum albumin (Sanquin), was loaded within the same activity range. The gel was exposed overnight to an imaging plate (BAS-MS; Fujifilm) and read with a Typhoon laser scanner (Amersham). Autoradiography on 4 µm tumor and spleen sections was performed similarly. The tissue slides were exposed with 1.5 µL of 1%, 0.1%, and 0.01% injected tracer on silica gel on thin-layer chromatography Al foils (Sigma-Aldrich). The maximum gray value uptake in tissue slides was converted into %ID per area based on the diluted tracer standards using ImageJ version 1.53k. The autoradiography slides were subsequently stained with hematoxylin and eosin to analyze tissue viability and morphology.

Supplemental References

1. Vosjan MJ, Perk LR, Visser GW, Budde M, Jurek P, Kiefer GE, et al. Conjugation and radiolabeling of monoclonal antibodies with zirconium-89 for PET imaging using the bifunctional chelate p-isothiocyanatobenzyl-desferrioxamine. Nat Protoc. 2010;5:739–43.
2. Verel I, Visser GW, Boellaard R, Stigter-van Walsum M, Snow GB, van Dongen GA. ^{89}Zr Immuno-PET: Comprehensive procedures for the production of ^{89}Zr-labeled monoclonal antibodies. J Nucl Med. 2003;44:1271–81.
3. Giesen D, Hooge MNL, Nijland M, Heyerdahl H, Dahle J, de Vries EGE, et al. ^{89}Zr-PET imaging to predict tumor uptake of ^{177}Lu-NNV003 anti-CD37 radioimmunotherapy in mouse models of B cell lymphoma. Sci Rep. 2022;12:6286.
4. Nagengast WB, de Vries EG, Hospers GA, Mulder NH, de Jong JR, Hollema H, et al. In vivo VEGF imaging with radiolabeled bevacizumab in a human ovarian tumor xenograft. J Nucl Med. 2007;48:1313–19.
5. Giesen D, Broer LN, Lub-de Hooge MN, et al. Probody therapeutic design of ^{89}Zr-CX-072 promotes accumulation in PD-L1–expressing tumors compared to normal murine lymphoid tissue. Clin Cancer Res 2020;26:3999–4009.
6. Giesen D, Broer LN, Lub-de Hooge MN, Popova I, Howng B, Nguyen M, et al. ^{89}Zr-atezolizumab imaging as a non-invasive approach to assess clinical response to PD-L1 blockade in cancer. Nat Med. 2018;24:1852–58.
7. Peper-Gabriel JK, Pavlidou M, Pattarini L, Morales-Kastresana A, Jaquin TJ, Gallou C, et al. The PD-L1/4-1BB bispecific antibody-Anticalin fusion protein PRS-

344/S095012 elicits strong T-cell stimulation in a tumor-localized manner. Clin Cancer Res. 2022;28:3387–99.

Supplemental Figures

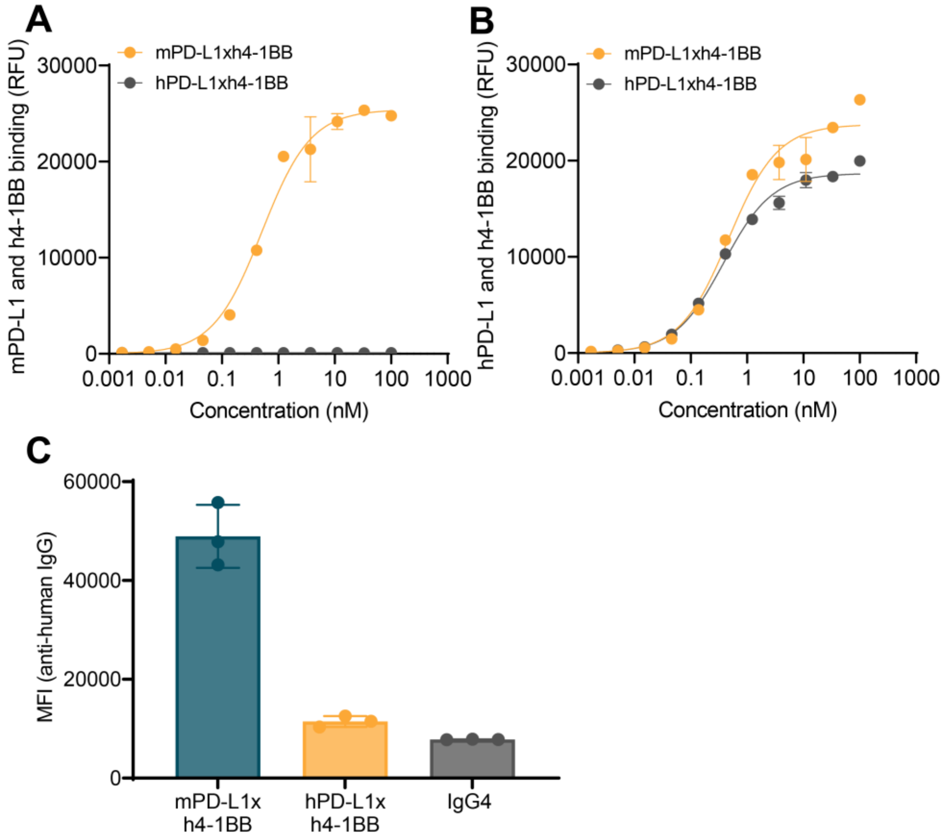

Figure S1. Characterization of mPD-L1xh4-1BB and hPD-L1xh4-1BB.

(A) Dual binding of mPD-L1xh4-1BB and hPD-L1xh4-1BB to recombinant murine PD-L1 and human 4-1BB assessed with ELISA. **(B)** Dual binding of mPD-L1xh4-1BB (cross-reactive to hPD-L1) and hPD-L1xh4-1BB to recombinant human PD-L1 and 4-1BB assessed with ELISA. **(C)** Binding of mPD-L1xh4-1BB, hPD-L1xh4-1BB, and IgG4 to murine PD-L1 positive MC38 tumor cells accessed with flow cytometry. Detection of antibodies was done with APC-anti-IgG (Jackson ImmunoResearch; 109-136-088). Data are presented as mean ± standard deviation

APC: allophycocyanin; ELISA: enzyme-linked immunosorbent assay; h: human; m: murine; MC38: murine colon adenocarcinoma cell line; MFI: mean fluorescent intensity; nM: nanomolar; RFU: relative fluorescent units.

A

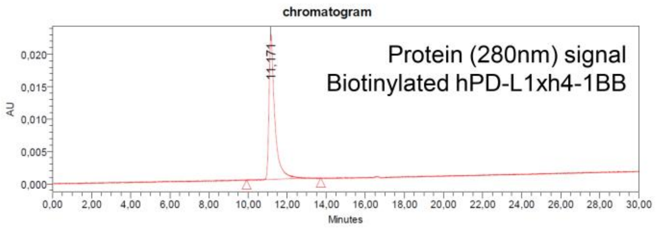

D

Relative potency (%)	human PD-L1	human 4-1BB
Biotinylated hPD-L1x h4-1BB	77.6%	30.5%

B C

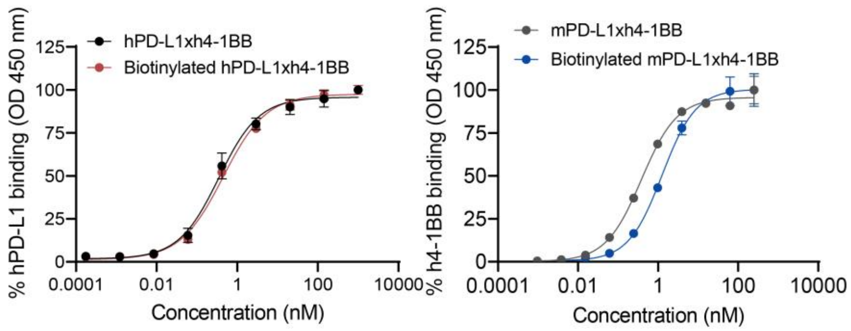

Figure S2. Characterization of biotinylated hPD-L1xh4-1BB.

(A) Size-exclusion ultra-high performance liquid chromatogram of the UV absorption at 280 nm of biotinylated hPD-L1xh4-1BB. **(B)** Binding of (biotinylated) hPD-L1xh4-1BB to recombinant human PD-L1 assessed by ELISA (n=3). **(C)** Binding of (biotinylated) hPD-L1xh4-1BB to recombinant human 4-1BB assessed by ELISA (n=3). In both ELISA experiments, the maximum specific concentration was similar between biotinylated and unconjugated Mabcalin. **(D)** Relative potencies of data presented in B and C.

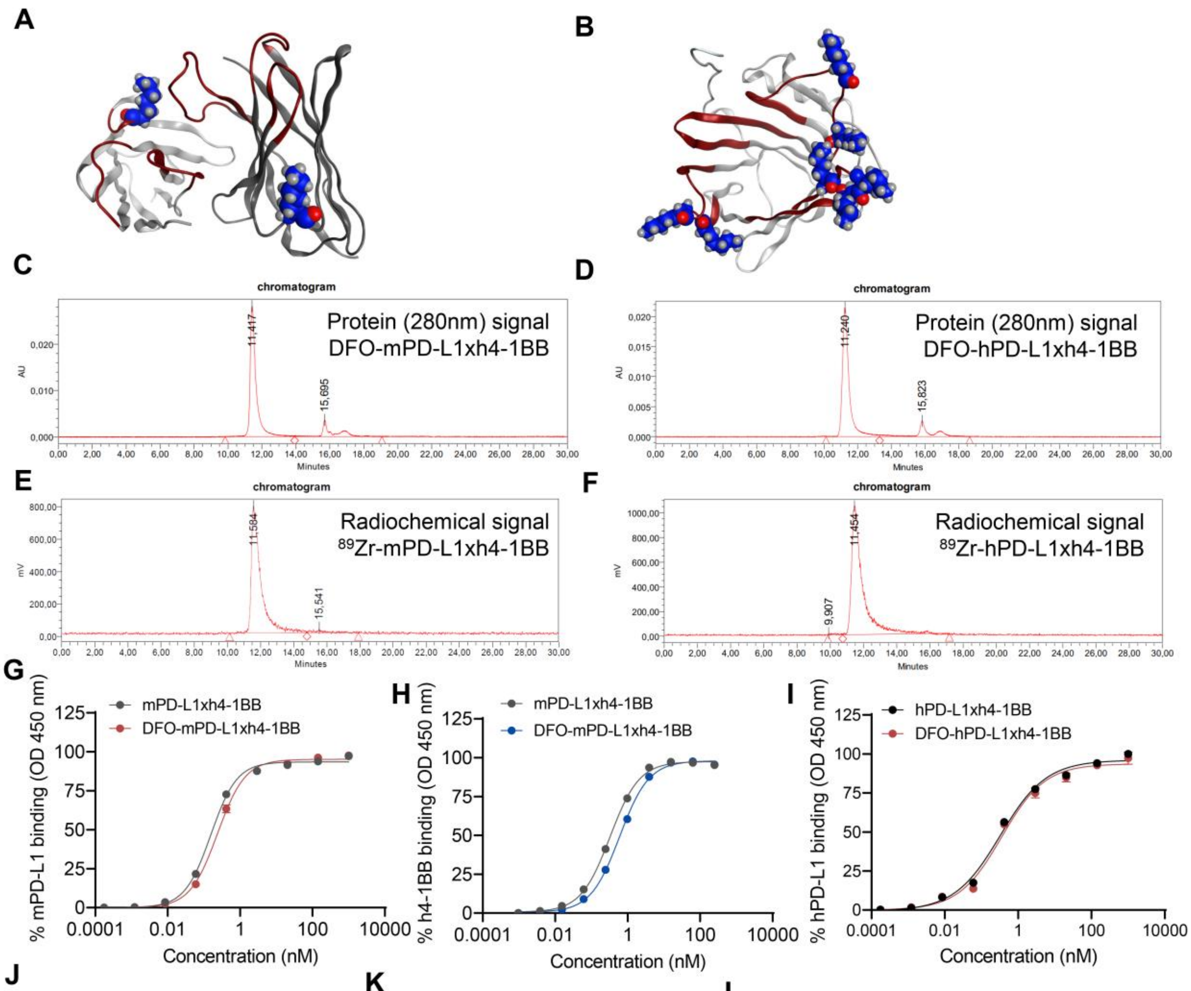

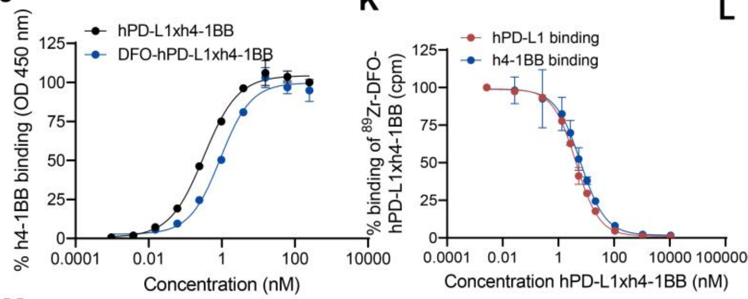

L

Relative potency (%)	human PD-L1	murine PD-L1	human 4-1BB
DFO-mPD-L1 x h4-1BB	NA	82.7%	53.3%
DFO-hPD-L1x h4-1BB	91.5%	NA	35.4%
^{89}Zr-hPD-L1x h4-1BB	116.5%	NA	80.1%

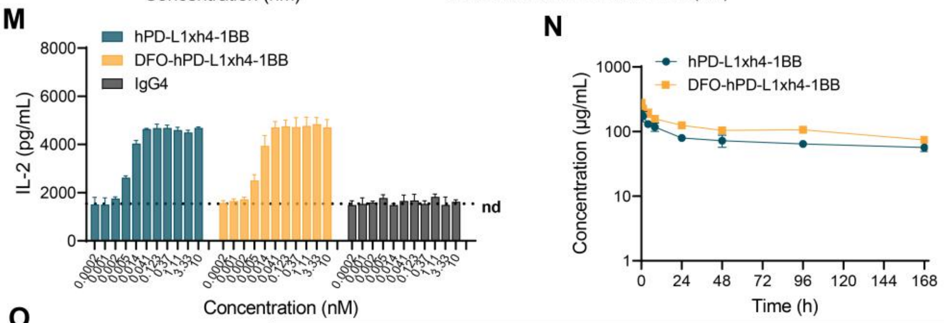

O

	AUC t = 168 h (ug*h/mL)	Half-life (h)	Clearance (mL/day/kg)
hPD-L1xh4-1BB	12163	344	6.0
DFO-hPD-L1xh4-1BB	18076	217	5.8

Figure S3. Characterization of DFO-conjugated and ^{89}Zr-radiolabeled mPD-L1xh4-1BB and hPD-L1xh4-1BB.

(A) Size-exclusion ultra-high performance liquid chromatogram of the UV absorption at 280 nm of biotinylated hPD-L1xh4-1BB. **(B)** Cartoon representation of the variable domain homology model of the hPD-L1xh4-1BB binding antibody portion of the Mabcalin. Complementarity-determining regions are shown in red, and constant regions of the variable fragment heavy and light chains are shown in dark and light grey, respectively. Surface-exposed lysine residues in the variable domain are shown as spheres with blue carbon atoms. **(C)** Cartoon representation of the Anticalin homology model of hPD-L1xh4-1BB. The four flexible regions of the Anticalin moiety involved in 4-1BB binding are shown in red, whereas the rest is in gray. Surface-exposed lysine residues are highlighted as in B. **(D)** Size-exclusion ultra-high performance liquid chromatogram of the UV absorption at 280 nm of DFO-mPD-L1xh4-1BB. **(E)** Size-exclusion ultra-high performance liquid chromatogram of the UV absorption at 280 nm of DFO-hPD-L1xh4-1BB. **(F)** Size-exclusion ultra-high performance liquid chromatogram of the ^{89}Zr-mPD-L1xh4-1BB radiochemical signal. **(G)** Size-exclusion ultra-high performance liquid chromatogram of the radiochemical signal of ^{89}Zr-hPD-L1xh4-1BB. **(H)** Binding of (DFO-) mPD-L1xh4-1BB to recombinant murine PD-L1 assessed by ELISA. **(I)** Binding of (DFO-) mPD-L1xh4-1BB to recombinant human 4-1BB assessed by ELISA. **(J)** Binding of (DFO-) hPD-L1xh4-1BB to recombinant human PD-L1 assessed by ELISA. Data from 3 individual experiments is presented with n = 3 per experiment. **(K)** Binding of (DFO-) hPD-L1xh4-1BB to recombinant human 4-1BB assessed by ELISA. Data from 3 individual experiments is presented with n=3 per experiment. In all ELISA experiments, the maximum specific

concentration was similar between DFO-conjugated and unconjugated Mabcalin. **(L)** Competitive binding affinity of ^{89}Zr-hPD-L1xh4-1BB compared to unmodified hPD-L1xh4-1BB. Data from 3 individual experiments is presented with n = 2 per experiment. **(M)** Relative potencies of data presented in H-K. **(N)** T-cell activation of peripheral blood mononuclear cells (PBMCs) measured as IL-2 secretion by staphylococcal enterotoxin B functional assay. The dashed line indicates IL-2 secretion of staphylococcal enterotoxin B-activated PBMCs without presented constructs. The result of one of the four tested donors is shown. **(O)** Plasma concentration-time profile following single intravenous administration of 10 mg/kg antibody into CD1 mice. Three mice per time point per group. **(P)** The area under the curve, half-life, and clearance of hPD-L1xh4-1BB and DFO-hPD-L1xh4-1BB in CD1 mice. Data are presented as mean ± standard deviation.

AUC: area under the curve; cpm: counts per min; h: human; IL-2: interleukin-2; m: murine; mV: microvolt; NA: not applicable; nd: not detected; nM: nanomolar; nm: nanometer; OD: optical density.

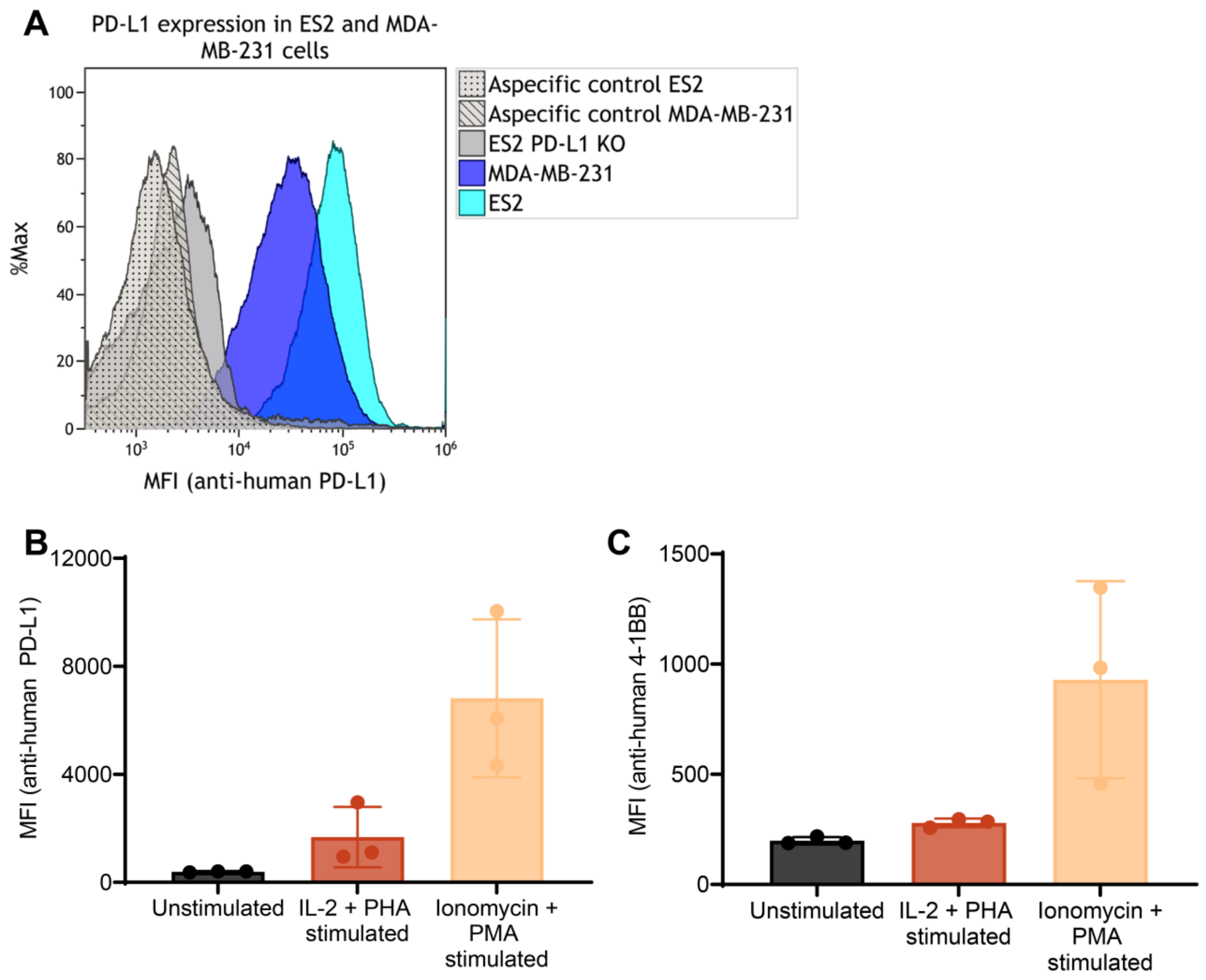

Figure S4. Receptor expression levels on MDA-MB-231, ES2, ionomycin + PMA, and IL-2 + PHA stimulated human T-cells.

(A) PD-L1 expression on MDA-MB-231, ES2 and ES2 PD-L1 knock-out cell lines. **(B)** PD-L1 expression of human T-cells of three individual PBMC donors after ionomycin + PMA activation for 16 h and IL-2 + PHA stimulation for 3 days. **(C)** 4-1BB expression of human T-cells of three individual PBMC donors after ionomycin/PMA stimulation for 16 h and IL-2/PHA activation for 3 days. Data are presented as mean ± standard deviation.

ES2 PD-L1 KO: PD-L1 knock-out cell line; IL-2: interleukin-2; MFI: mean fluorescent intensity; PBMCs: peripheral blood mononuclear cells; PHA: lectin from Phaseolus vulgaris; PMA: phorbol myristate acetate.

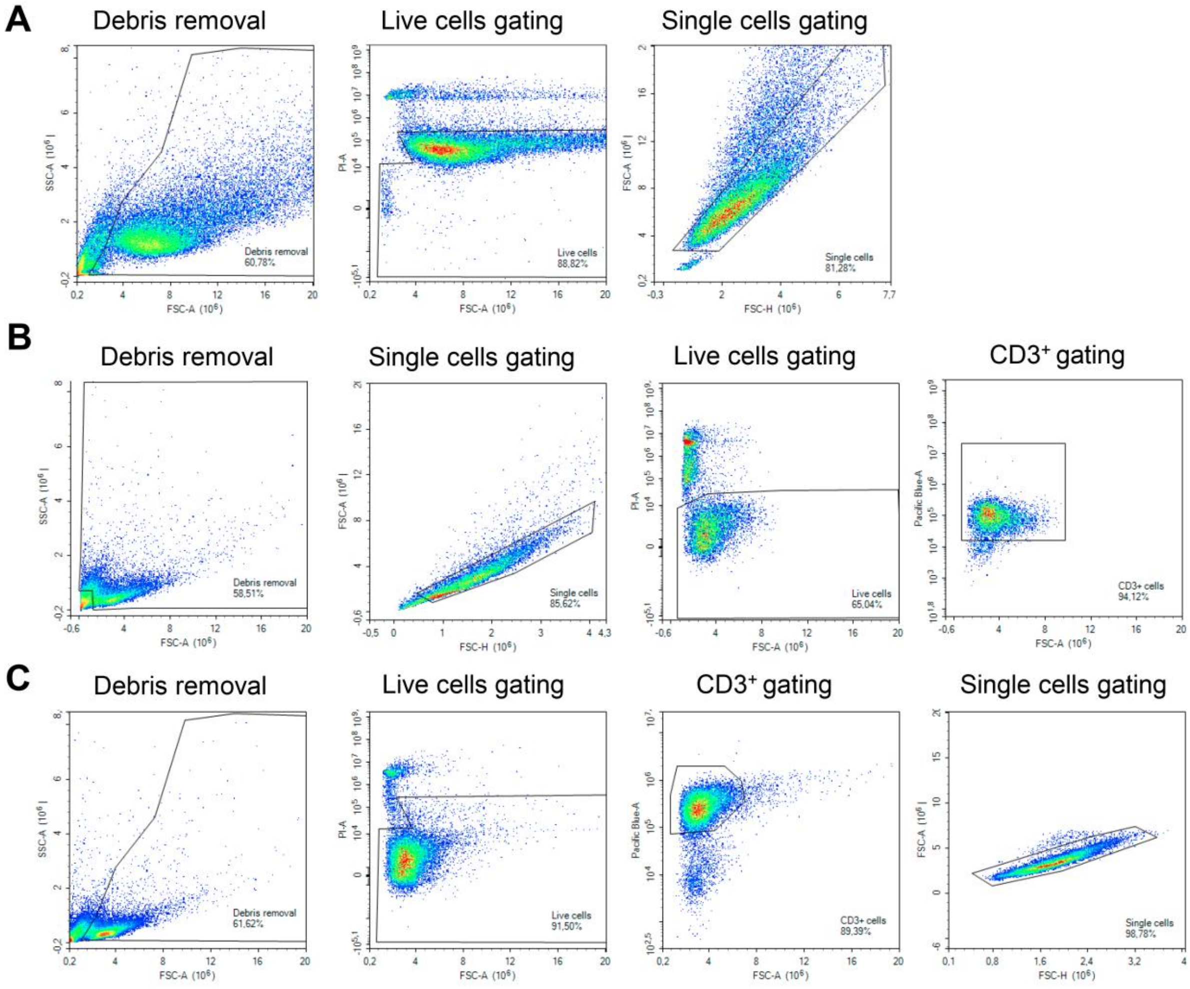

Figure S5. Flow cytometry gating strategy used in internalization experiments.

(A) Gating strategy for selecting healthy single tumor cells. **(B)** Gating strategy for selecting healthy single T-cells (CD3-positive cells) from PBMCs stimulated with ionomycin/PMA. **(C)** Gating strategy for selecting healthy single human T-cells from PBMCs stimulated with IL-2/PHA. Debris was removed in an FSC versus an SSC dot plot. Single cells were gated by plotting FSC-H versus area FSC-A, and live cells were gated based on a PI-A staining. CD3-positive human cells in the PBMC samples were gated on the anti-human Brilliant Violet^{TM} 421 (BV421) CD3 staining and measured in the Pacific Blue-A. Binding/internalization of biotinylated hPD-Lxh4-1BB Mabcalin was detected

using Alexa Fluor™ 647 conjugated streptavidin within the live single tumor and T-cells and expressed as MFI. Samples were measured in triplicate and corrected for background fluorescence.

FSC: forward scatter; FSC-A: forward scatter area; FSC-H: forward scatter height; IL-2: interleukin 2; MFI: mean fluorescent intensity; Pacific Blue-A: pacific blue area; PBMCs: peripheral blood mononuclear cells; PHA: phytohemagglutinin; PI-A: propidium iodide area; PMA: phorbol myristate acetate; SSC: side scatter; SSC-A: side scatter area.

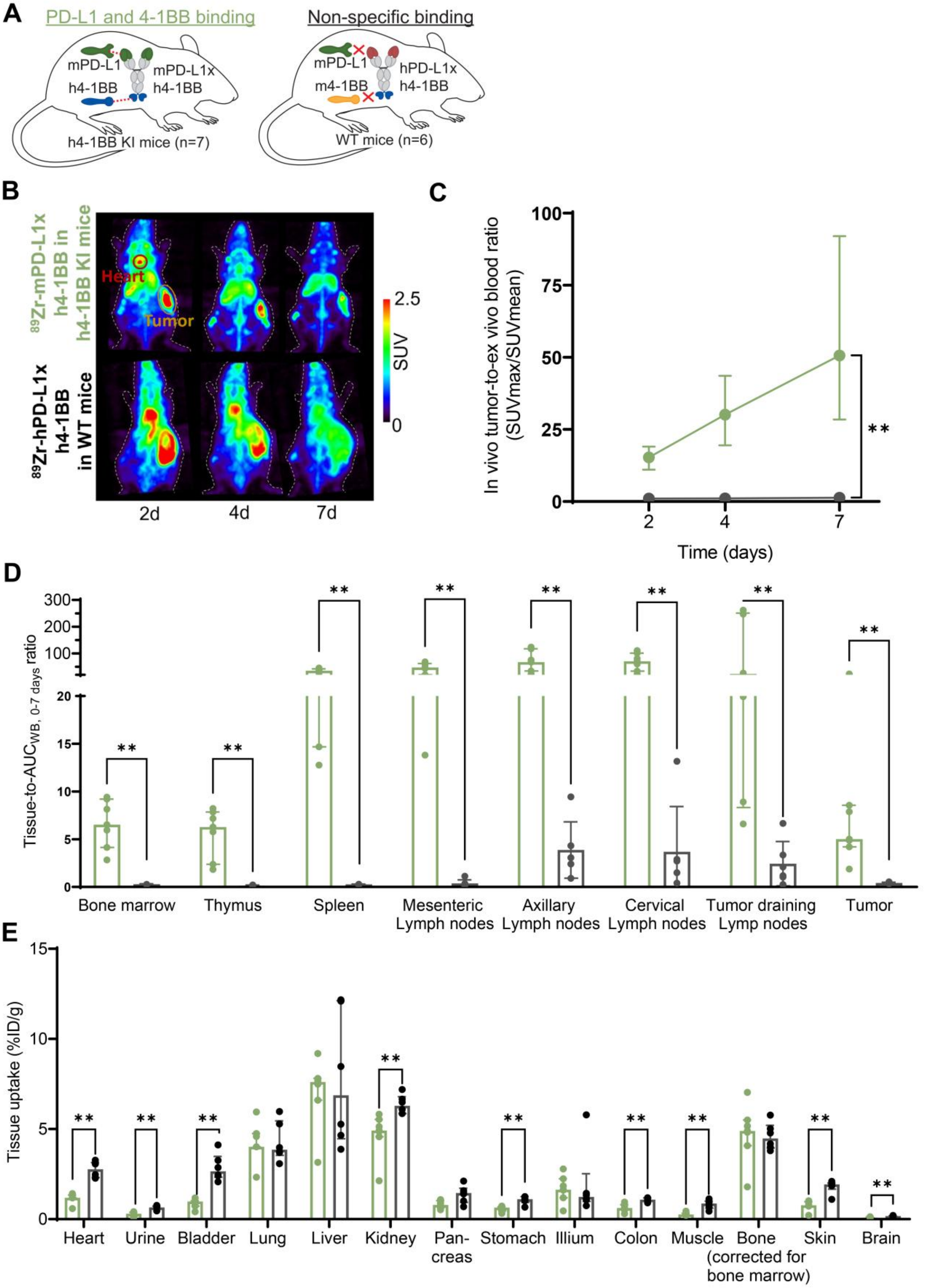
A
PD-L1 and 4-1BB binding
mPD-L1
mPD-L1x
h4-1BB
h4-1BB
h4-1BB KI mice (n=7)
Non-specific binding
mPD-L1
hPD-L1x
m4-1BB
h4-1BB
WT mice (n=6)
B
89Zr-mPD-L1x h4-1BB in h4-1BB KI mice
89Zr-hPD-L1x h4-1BB in WT mice
Heart
Tumor
2.5
SUV
0
2d
4d
7d
C
In vivo tumor-to-ex vivo blood ratio (SUVmax/SUVmean)
100
75
50
25
0
**
2
4
7
Time (days)
D
Tissue-to-AUCWB, 0-7 days ratio
300
200
100
20
15
10
5
0
**
Bone marrow
Thymus
Spleen
Mesenteric Lymph nodes
Axillary Lymph nodes
Cervical Lymph nodes
Tumor draining Lymp nodes
Tumor
E
Tissue uptake (%ID/g)
15
10
5
0
**
Heart
Urine
Bladder
Lung
Liver
Kidney
Pan-creas
Stomach
Illium
Colon
Muscle
Bone (corrected for bone marrow)
Skin
Brain

Figure S6. Specificity of ^{89}Zr-mPD-L1xh4-1BB in MC38-tumor bearing h4-1BB KI mice dosed at 30 µg.

(A) Overview of experimental groups. Both Mabcalins are dosed at 30 µg. **(B)** Maximum intensity PET images of MC38 tumor-bearing h4-1BB KI or WT mice given 30 µg of ^{89}Zr-mPD-L1xh4-1BB or ^{89}Zr-hPD-L1xh4-1BB at 2, 4, and 7 days post-injection. A red circle highlights the heart, and a yellow circle the tumor. **(C)** In vivo tumor-to-ex vivo blood ratios at days 2, 4, and 7 post-injection. Ex vivo blood SUV was calculated based on blood samples after the PET scan. **(D)** Ex vivo uptake (tissue-to-$AUC_{WB,\ 0\text{-}7\ days}$) 7 days post-injection in lymphoid tissues and the tumor. **(E)** Ex vivo uptake (%ID/g) 7 days post-injection in organs in non-lymphoid tissues. Data are presented as median ± interquartile range.

%ID/g: percentage injected dose per gram tissue; $AUC_{WB,\ 0\text{-}7\ days}$: whole blood area under the curve from 0 to 7 days; d: day; h: human; h4-1BB KI mice: human 4-1BB knock-in mice; m: murine; SUV: standard uptake value; WT mice: wild-type mice; *: $P \leq 0.05$; **: $P \leq 0.01$.

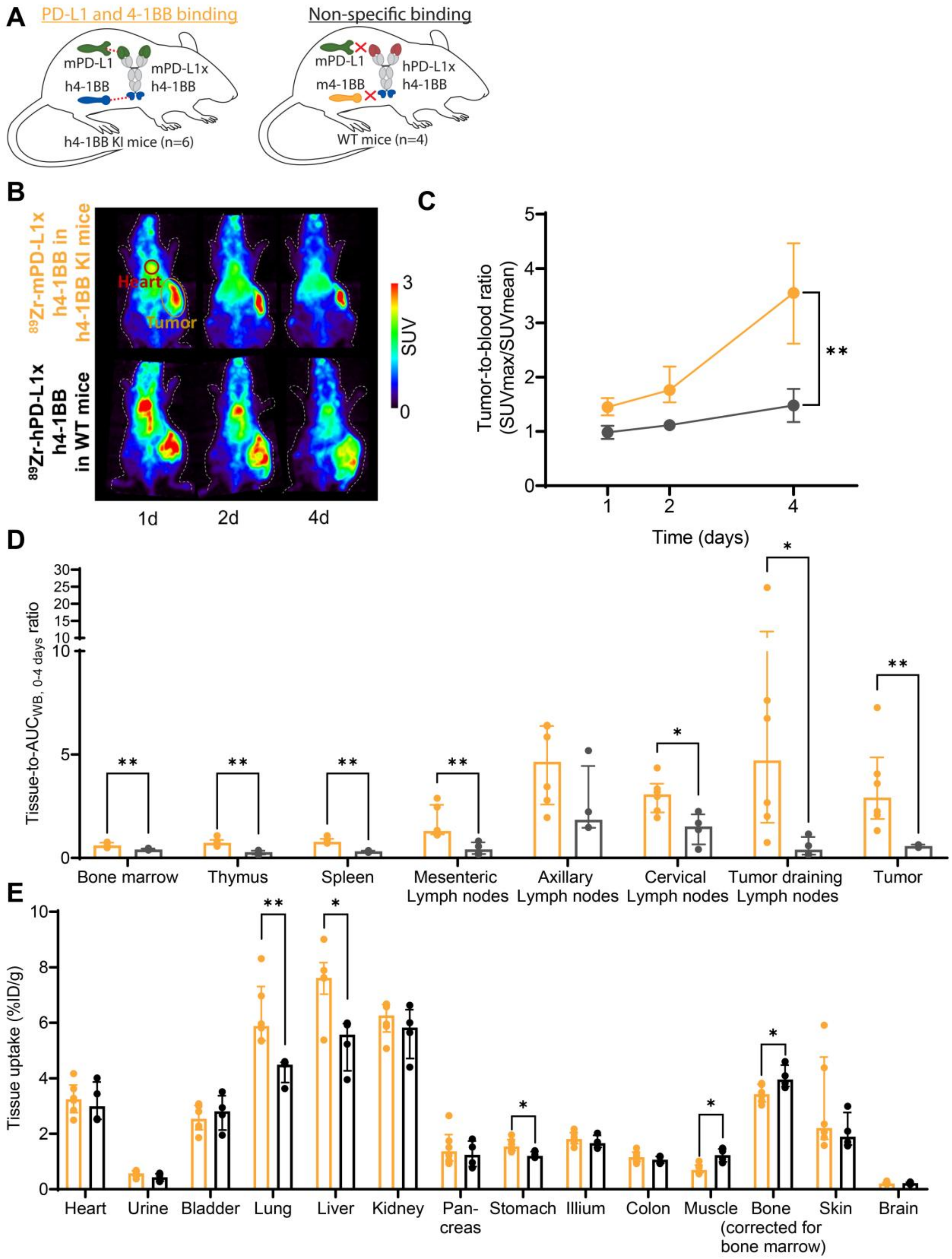
A
PD-L1 and 4-1BB binding
mPD-L1
h4-1BB
mPD-L1x
h4-1BB
h4-1BB KI mice (n=6)
Non-specific binding
mPD-L1
m4-1BB
hPD-L1x
h4-1BB
WT mice (n=4)
B
^{89}Zr-mPD-L1x h4-1BB in h4-1BB KI mice
^{89}Zr-hPD-L1x h4-1BB in WT mice
Heart
Tumor
1d
2d
4d
SUV
C
Tumor-to-blood ratio (SUVmax/SUVmean)
Time (days)
D
Tissue-to-AUC$_{WB, 0-4 days}$ ratio
Bone marrow
Thymus
Spleen
Mesenteric Lymph nodes
Axillary Lymph nodes
Cervical Lymph nodes
Tumor draining Lymph nodes
Tumor
E
Tissue uptake (%ID/g)
Heart
Urine
Bladder
Lung
Liver
Kidney
Pan-creas
Stomach
Illium
Colon
Muscle
Bone (corrected for bone marrow)
Skin
Brain

Figure S7. Specificity of ^{89}Zr-mPD-L1xh4-1BB in MC38 tumor-bearing h4-1BB KI mice dosed at 200 µg.

(A) Overview of experimental groups. Both Mabcalins are dosed at 200 µg. **(B)** Maximum intensity PET images of MC38 tumor-bearing h4-1BB KI or WT mice given 200 µg of ^{89}Zr-mPD-L1xh4-1BB or ^{89}Zr-hPD-L1xh4-1BB at 1, 2, and 4 days post-injection. A red circle highlights the heart, and a yellow circle the tumor. **(C)** In vivo uptake in tumor-to-blood ratios at days 1, 2, and 4 post-injection. **(D)** Ex vivo uptake (tissue-to-$AUC_{WB,\ 0\text{-}4\ days}$) 4 days post-injection in lymphoid tissues and the tumor. **(E)** Ex vivo uptake (%ID/g) 4 days post-injection in non-lymphoid tissues. Data are presented as median ± interquartile range.

%ID/g: percentage injected dose per gram tissue; $AUC_{WB,\ 0\text{-}4\ days}$: whole blood area under the curve from 0 to 4 days; d: day; h: human; h4-1BB KI: human 4-1BB knock-in mice; m: murine; SUV: standard uptake value; WT mice: wild-type mice; *: $P \leq 0.05$; **: $P \leq 0.01$.

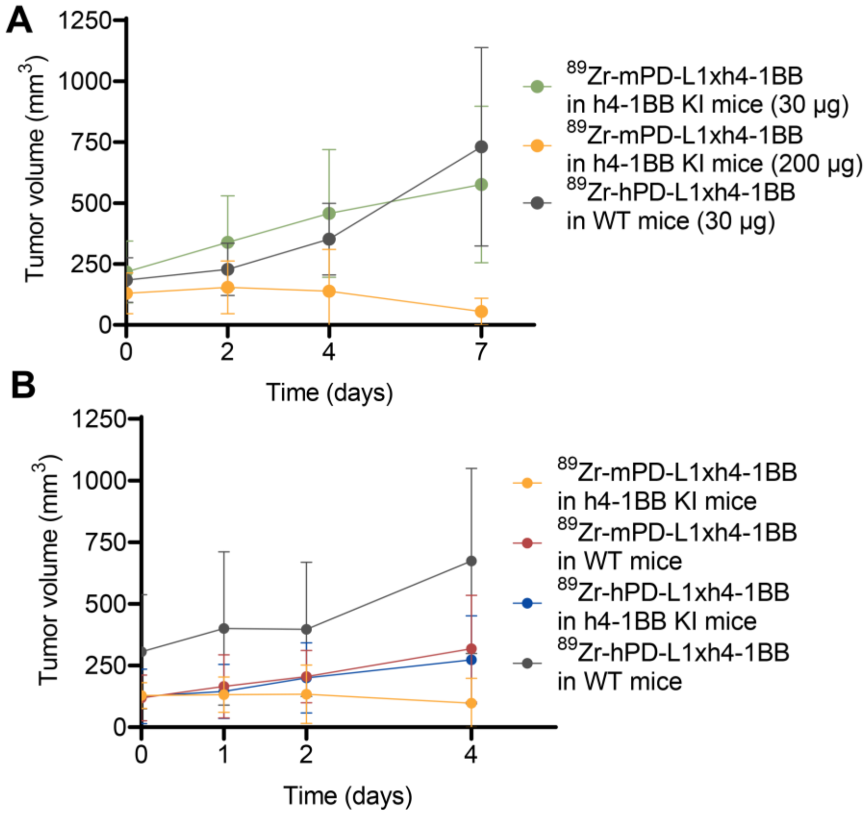

Figure S8. Tumor size during the imaging time frame.

(A) MC38 tumor volume in h4-1BB KI or WT mice that received ^{89}Zr-mPD-L1xh4-1BB or ^{89}Zr-hPD-L1xh4-1BB with a total protein dose of 30 or 200 µg at days 0, 2, 4, and 7 post-injection. **(B)** MC38 tumor volume in h4-1BB KI or WT mice given ^{89}Zr-mPD-L1xh4-1BB or ^{89}Zr-hPD-L1xh4-1BB with a total protein dose of 200 µg at days 0, 1, 2, and 4 post-injection. Data are presented as mean ± standard deviation.

H: human; h4-1BB KI mice: human 4-1BB knock-in mice; m: murine; WT mice: wild-type mice.

Table S1. In vivo tumor uptake (SUVmax) and in vivo tumor-to-ex vivo blood ratio (SUVmax/SUVmean) of ^{89}Zr-mPD-L1x4-1BB and ^{89}Zr-hPD-L1x4-1BB in h4-1BB KI or WT mice at 2, 4 and 7 days post-injection.

Data are presented as median ± interquartile range.

Time (days)		^{89}Zr-mPD-L1xh4-1BB in h4-1BB KI mice (30 µg; n=7)	^{89}Zr-mPD-L1xh4-1BB in h4-1BB KI mice (200 µg; n=5)	^{89}Zr-hPD-L1xh4-1BB in WT mice (30 µg; n=6)
2	Tumor	2.58 (1.86 – 3.66)	2.83 (2.50 – 3.75)	NA
	In vivo tumor-to-ex vivo blood ratio	15.29 (11.06 – 19.02)	NA	1.08 (0.84 – 1.19)
4	Tumor	1.57 (1.09 – 3.06)	3.67 (2.97 – 5.33)	NA
	In vivo tumor-to-ex vivo blood ratio	30.09 (19.50 – 43.59)	NA	1.11 (1.01 – 1.15)
7	Tumor	1.20 (0.74 – 2.23)	4.99 (3.71 – 8.23)	NA
	In vivo tumor-to-ex vivo blood ratio	50.63 (28.45 – 92.04)	NA	1.31 (1.15 – 1.46)

NA, not applicable

Table S2. In vivo tumor-to-blood ratio (SUVmax/SUVmean) of ^{89}Zr-mPD-L1x4-1BB and ^{89}Zr-hPD-L1x4-1BB in h4-1BB KI or WT mice at 1, 2 and 4 days post-injection.

Data are presented as median ± interquartile range.

Time (days)	^{89}Zr-mPD-L1xh4-1BB in h4-1BB KI mice (200 µg; n=6)	^{89}Zr-mPD-L1xh4-1BB in WT mice (200 µg; n=4)	^{89}Zr-hPD-L1xh4-1BB in 4-BB KI mice (200 µg; n=5)	^{89}Zr-hPD-L1xh4-1BB in WT mice (200 µg; n=4)
1	1.45 (1.30 – 1.61)	0.94 (0.78 – 1.16)	0.98 (0.83 – 1.20)	0.97 (0.88 – 1.11)
2	1.76 (1.54 – 2.19)	1.15 (0.94 – 1.28)	1.25 (1.06 – 1.40)	1.09 (1.03 – 1.23)
4	3.55 (2.62 – 4.47)	1.64 (1.40 – 2.01)	1.41 (1.35 – 1.55)	1.48 (1.18 – 1.78)

Table S3. Absolute ex vivo uptake (%ID/g) of ^{89}Zr-mPD-L1x4-1BB and ^{89}Zr-hPD-L1x4-1BB in h4-1BB KI or WT mice at 7 days post-injection in lymphoid tissues and the tumor.

Data are presented as median ± interquartile range.

Tissue	^{89}Zr-mPD-L1xh4-1BB in h4-1BB KI mice (30 µg; n=7)	^{89}Zr-mPD-L1xh4-1BB in h4-1BB KI mice (200 µg; n=5)	^{89}Zr-hPD-L1xh4-1BB in WT mice (30 µg; n=6)
**Bone marrow**	2.72 (1.82 – 3.09)	3.48 (2.81 – 5.07)	4.10 (3.63 – 5.31)
**Thymus**	2.40 (2.12 – 2.67)	4.22 (3.85 – 5.72)	2.38 (1.44 – 2.97)
**Spleen**	12.59 (10.40 – 15.62)	8.98 (6.98 – 10.56)	4.04 (3.48 – 5.05)
**Mesentric lymph nodes**	17.58 (14.59 – 24.45)	10.11 (7.74 – 12.67)	3.72 (2.89 – 8.07)
**Axillary lymph nodes**	28.50 (20.13 – 40.26)	32.15 (12.17 – 45.36)	54.14 (27.77 – 82.23)
**Cervical lymph nodes**	32.97 (18.47 – 38.32)	20.75 (14.49 – 73.37)	31.50 (18.70 – 96.29)
**Tumor draining lymph nodes**	6.82 (2.67 – 83.30)	16.48 (7.36 – 36.33)	26.02 (11.41 – 66.81)
**Tumor**	2.53 (1.53 – 5.12)	19.03 (10.27 – 54.09)	6.11 (5.35 – 7.73)

Table S4. Ex vivo uptake (tissue-to-blood $AUC_{WB,\ 0\text{-}7\ days}$) of ^{89}Zr-mPD-L1x4-1BB and ^{89}Zr-hPD-L1x4-1BB in h4-1BB KI or WT mice at 7 days post-injection in lymphoid tissues and the tumor.

Data are presented as median ± interquartile range.

Tissue	^{89}Zr-mPD-L1xh4-1BB in h4-1BB KI mice (30 µg; n=7)	^{89}Zr-mPD-L1xh4-1BB in h4-1BB KI mice (200 µg; n=5)	^{89}Zr-hPD-L1xh4-1BB in WT mice (30 µg; n=6)
**Bone marrow**	6.52 (4.14 – 9.20)	0.63 (0.62 – 0.70)	0.28 (0.23 – 0.33)
**Thymus**	6.28 (2.38 – 7.86)	0.87 (0.71 – 0.89)	0.14 (0.10 – 0.21)
**Spleen**	35.66 (14.68 – 42.59)	1.48 (1.07 -2.18)	0.28 (0.23 – 0.30)
**Mesentric lymph nodes**	47.77 (20.05 – 62.26)	1.71 (1.24 – 2.68)	0.25 (0.16 – 0.54)
**Axillary lymph nodes**	67.63 (34.64 – 117.31)	3.47 (2.58 – 8.31)	3.07 (2.04 – 5.60)
**Cervical lymph nodes**	70.90 (33.79 – 101.06)	4.10 (3.03 – 8.77)	2.11 (1.19 – 5.46)
**Tumor draining lymph nodes**	23.25 (8.33 – 251.01)	4.06 (1.22 – 6.21)	1.64 (0.84 – 4.18)
**Tumor**	5.01 (4.20 – 8.56)	2.93 (2.22 – 9.24)	0.42 (0.37 – 0.46)

Table S5. Absolute ex vivo uptake (%ID/g) of ^{89}Zr-mPD-L1x4-1BB and ^{89}Zr-hPD-L1x4-1BB in h4-1BB KI or WT mice at 4 days post-injection in lymphoid tissues and the tumor.

Data are presented as median ± interquartile range.

Tissue	^{89}Zr-mPD-L1xh4-1BB in h4-1BB KI mice (200 µg; n=6)	^{89}Zr-mPD-L1xh4-1BB in WT mice (200 µg; n=4)	^{89}Zr-hPD-L1xh4-1BB in 4-BB KI mice (200 µg; n=5)	^{89}Zr-hPD-L1xh4-1BB in WT mice (200 µg; n=4)
**Bone marrow**	4.44 (3.74 – 5.30)	4.50 (4.18 – 5.56)	5.07 (4.52 – 5.58)	4.86 (4.14 – 5.62)
**Thymus**	5.13 (4.75 – 5.66)	4.58 (4.24 – 5.09)	3.19 (2.96 – 4.61)	3.56 (1.93 – 3.79)
**Spleen**	5.51 (4.98 – 6.01)	5.22 (4.76 – 6.03)	3.27 (3.14 – 4.35)	3.84 (3.01 – 4.30)
**Mesentric lymph nodes**	9.69 (8.18 – 13.97)	4.48 (2.49 – 8.05)	4.30 (3.66 – 13.92)	4.36 (2.33 – 9.08)
**Axillary lymph nodes**	27.69 (17.87 – 44.21)	19.41 (12.48 – 25.11)	51.31 (32.24 – 101.3)	21.00 (16.80 – 54.24)
**Cervical lymph nodes**	20.40 (14.42 – 25.75)	14.45 (12.60 – 33.75)	26.44 (18.70 – 42.19)	16.33 (7.67 – 25.98)
**Tumor draining lymph nodes**	32.80 (8.49 – 87.30)	6.05 (3.52 – 9.82)	12.00 (6.91 – 15.86)	4.80 (1.67 – 12.96)
**Tumor**	21.13 (10.19 – 34.56)	7.39 (6.15 – 8.43)	9.72 (7.20 – 11.32)	6.84 (5.39 – 7.79)

Table S6. Ex vivo uptake (tissue-to-blood $AUC_{WB,\ 0\text{-}4\ days}$) of ^{89}Zr-mPD-L1x4-1BB and ^{89}Zr-hPD-L1x4-1BB in h4-1BB KI or WT mice at 4 days post-injection in lymphoid tissues and the tumor.

Data are presented as median ± interquartile range.

Tissue	^{89}Zr-mPD-L1xh4-1BB in h4-1BB KI mice (200 µg; n=6)	^{89}Zr-mPD-L1xh4-1BB in WT mice (200 µg; n=4)	^{89}Zr-hPD-L1xh4-1BB in 4-BB KI mice (200 µg; n=5)	^{89}Zr-hPD-L1xh4-1BB in WT mice (200 µg; n=4)
**Bone marrow**	0.61 (0.57 – 0.74)	0.55 (0.51 – 0.60)	0.42 (0.41 – 0.45)	0.41 (0.38 – 0.46)
**Thymus**	0.73 (0.66 – 0.87)	0.54 (0.48 – 0.60)	0.30 (0.26 – 0.36)	0.28 (0.17 – 0.35)
**Spleen**	0.78 (0.70 – 0.92)	0.59 (0.54 – 0.75)	0.29 (0.28 – 0.34)	0.32 (0.28 – 0.35)
**Mesentric lymph nodes**	1.30 (1.14 – 2.57)	0.48 (0.30 – 1.00)	0.33 (0.32 – 1.25)	0.41 (0.19 – 0.75)
**Axillary lymph nodes**	4.65 (2.58 – 6.38)	2.21 (1.59 – 2.77)	4.70 (2.84 – 8.01)	1.85 (1.46 – 4.45)
**Cervical lymph nodes**	3.07 (2.20 – 3.59)	1.61 (1.46 – 4.22)	2.45 (1.56 – 3.55)	1.52 (0.65 – 2.11)
**Tumor draining lymph nodes**	4.71 (1.70 – 11.91)	0.64 (0.42 – 1.22)	1.05 (0.62 – 1.26)	0.40 (0.16 – 1.02)
**Tumor**	2.92 (1.88 – 4.86)	0.84 (0.78 – 0.91)	0.72 (0.64 – 0.99)	0.57 (0.49 – 0.65)

Table S7. Ex vivo uptake (tissue-to-blood $AUC_{WB,\ 0\text{-}7\ days}$) of ^{89}Zr-mPD-L1x4-1BB and ^{89}Zr-hPD-L1x4-1BB in h4-1BB KI or WT mice at 7 days post-injection in non-lymphoid tissues.

Data are presented as median ± interquartile range.

Tissue	^{89}Zr-mPD-L1xh4-1BB in h4-1BB KI mice (30 µg; n=7)	^{89}Zr-mPD-L1xh4-1BB in h4-1BB KI mice (200 µg;n=5)	^{89}Zr-hPD-L1xh4-1BB in WT mice (30 µg; n=6)
**Heart**	1.19 (1.18 – 1.34)	2.24 (1.56 – 2.67)	2.77 (2.31 – 3.14)
**Urine**	0.30 (0.17 – 0.38)	0.60 (0.48 – 1.10)	0.65 (0.55 – 0.72)
**Bladder**	0.98 (0.72 – 1.02)	1.61 (0.92 – 1.91)	2.66 (2.33 – 3.48)
**Lung**	4.01 (4.01 – 4.75)	5.75 (3.88 – 6.38)	3.86 (3.55 – 5.45)
**Liver**	7.61 (6.60 – 7.81)	9.76 (8.69 – 10.96)	6.87 (4.46 – 12.12)
**Kidney**	4.92 (4.57 – 5.53)	5.83 (4.73 – 6.11)	6.29 (5.86 – 6.79)
**Pancreas**	0.79 (0.64 – 1.03)	0.72 (0.62 – 0.88)	1.45 (0.93 – 1.71)
**Stomach**	0.64 (0.45 – 0.68)	0.98 (0.63 – 1.28)	1.11 (0.93 – 1.25)
**Illium**	1.63 (1.19 – 2.24)	1.78 (1.07 – 2.07)	1.24 (0.99 – 2.52)
**Colon**	0.62 (0.51 – 0.80)	0.81 (0.72 – 1.03)	1.09 (1.00 – 1.18)
**Muscle**	0.26 (0.21 – 0.32)	0.60 (0.32 – 0.74)	0.87 (0.61 – 1.00)
**Bone (corrected for bone marrow)**	4.89 (4.08 – 5.50)	5.21 (4.08 – 6.05)	4.48 (3.95 – 5.21)
**Skin**	0.77 (0.72 – 0.97)	0.92 (0.75 – 1.56)	1.93 (1.66 – 2.00)
**Brain**	0.08 (0.07 – 0.12)	0.15 (0.12 – 0.19)	0.17 (0.15 – 0.19)

Table S8. Ex vivo uptake (tissue-to-blood $AUC_{WB,\ 0\text{-}7\ days}$) of ^{89}Zr-mPD-L1x4-1BB and ^{89}Zr-hPD-L1x4-1BB in h4-1BB KI or WT mice at 4 days post-injection in non-lymphoid tissues.

Data are presented as median ± interquartile range.

Tissue	^{89}Zr-mPD-L1xh4-1BB in h4-1BB KI mice (200 µg; n=6)	^{89}Zr-mPD-L1xh4-1BB in WT mice (200 µg; n=4)	^{89}Zr-hPD-L1xh4-1BB in 4-BB KI mice (200 µg; n=5)	^{89}Zr-hPD-L1xh4-1BB in WT mice (200 µg; n=4)
**Heart**	3.24 (2.76 – 3.76)	2.68 (2.40 – 2.75)	3.01 (2.68 – 4.53)	2.99 (2.52 – 3.87)
**Urine**	0.58 (0.48 – 0.67)	0.49 (0.37 – 0.52)	0.32 (0.29 – 0.70)	0.43 (0.30 – 0.56)
**Bladder**	2.54 (2.15 – 3.02)	2.95 (2.80 – 3.04)	2.90 (2.72 – 4.98)	2.81 (2.13 – 3.37)
**Lung**	5.89 (5.36 – 7.31)	4.63 (4.23 – 5.50)	5.17 (4.33 – 5.68)	4.49 (3.84 – 4.58)
**Liver**	7.62 (7.03 – 8.17)	8.31 (6.51- 11.10)	6.90 (5.84 – 7.25)	5.58 (4.27 – 5.97)
**Kidney**	6.27 (5.67 – 6.66)	5.66 (5.29 – 6.22)	6.33 (4.73 – 7.01)	5.83 (4.71 – 6.47)
**Pancreas**	1.37 (1.01 – 1.97)	0.93 (0.82 – 0.93)	1.77 (0.99 – 2.13)	1.24 (0.82 – 1.74)
**Stomach**	1.54 (1.39 – 1.79)	1.42 (1.36 – 1.83)	1.62 (1.14 – 1.79)	1.21 (1.15 – 1.36)
**Illium**	1.81 (1.64 – 2.05)	1.91 (1.58 – 2.33)	1.63 (1.39 – 1.87)	1.66 (1.51 – 1.94)
**Colon**	1.16 (1.00 – 1.34)	1.11 (1.06 – 1.53)	1.23 (0.96 – 1.78)	1.07 (0.93 – 1.18)
**Muscle**	0.70 (0.60 – 0.87)	0.78 (0.49 – 1.47)	0.90 (0.77 – 1.37)	1.23 (0.99 – 1.45)
**Bone (corrected for bone marrow)**	3.43 (3.15 – 3.78)	3.23 (2.87 – 3.60)	3.56 (2.66 – 4.00)	3.96 (3.70 – 4.47)
**Skin**	2.21 (1.80 – 4.76)	2.47 (2.32 – 2.76)	2.59 (2.26 – 4.86)	1.90 (1.60 – 2.77)
**Brain**	0.21 (0.18 – 0.24)	0.20 (0.17 – 0.23)	0.20 (0.18 – 0.24)	0.22 (0.18 – 0.25)
